# Supplementary material for: EZH2 mutations in follicular lymphoma distort H3K27me3 profiles and alter transcriptional responses to PRC2 inhibition
Source: Nat Commun. 2024 Apr 24;15:3452. doi: 10.1038/s41467-024-47701-x (PMC11043461; doi:10.1038/s41467-024-47701-x)

Supplementary Information
(6 Tables, 5 Figures)

for

EZH2 mutations in follicular lymphoma distort H3K27me3 profiles and alter transcriptional responses to PRC2 inhibition

Pierre Romero[1,2,6], Laia Richart[1,6], Setareh Aflaki[1], Ambre Petitalot[1], Megan Burton[1], Audrey Michaud[1], Julien Masliah-Planchon[3], Frédérique Kuhnowski[4], Samuel Le Cam[1], Carlos Baliñas-Gavira[1], Céline Méaudre[2], Armelle Luscan[1], Abderaouf Hamza[3], Patricia Legoix[5], Anne Vincent-Salomon[2], Michel Wassef[1], Daniel Holoch[1]# & Raphaël Margueron[1]#

[1] Institut Curie, INSERM U934/CNRS UMR 3215, Paris Sciences et Lettres Research University, Sorbonne University, Paris, France
[2] Institut Curie, Department of Pathology, Paris Sciences et Lettres Research University, Paris, France
[3] Institut Curie, Pharmacogenetics Unit, Department of Genetics, Paris Sciences et Lettres Research University, Paris, France
[4] Institut Curie, Department of Clinical Hematology, Paris Sciences et Lettres Research University, Paris, France
[5] Institut Curie Genomics of Excellence (ICGex) Platform, Paris Sciences et Lettres Research University, Paris, France
[6] These authors contributed equally: Pierre Romero, Laia Richart
# e-mail: daniel.holoch@curie.fr; raphael.margueron@curie.fr

Supplementary Table 1. Scaling factors computed for H3K27me3 CUT&RUN-seq on WT and *Ezh2*$^{Y641F/WT}$ iMEFs presented in Fig. 2d and Supplementary Fig. 2c,e.

NGS sample number	Genotype	Total reads	*M. musculus* reads	*D. melanogaster* reads	Scaling factor (csaw)	Scaling factor (spike-in)
D127C53	WT	41 486 896	41 258 918	214 322	1.604102	1.16242
D127C54	WT	46 194 320	45 935 832	242 484	1.487783	1.0181
D127C57	*Ezh2*$^{Y641F/WT}$	78 910 504	78 667 180	226 088	0.7959	1.1025
D127C58	*Ezh2*$^{Y641F/WT}$	59 173 698	58 970 964	188 702	0.7366	1.3220

Supplementary Table 2. Demographic and disease characteristics of patients in the initial cohort as a function of *EZH2* status.

	***EZH2* WT**	***EZH2* mutant**
	**(*n* = 129)**	**(*n* = 31)**
**Age at diagnosis –yrs**		
Median	63.4	60.8
Range	28-87	37-65
**Sex –no. (%)**		
Female	80 (62)	21 (68)
Male	49 (38)	10 (32)
**Death –no. (%) ***	40 (31)	9 (29)
**Histological transformation –no. (%) ***	30 (18)	4 (13)

* Patients with no reported death or histological transformation include those whose follow-up information was unavailable. Last follow-up for WT cases not included in the restricted cohort is December 2019. Last follow-up for mutant cases, irrespective of inclusion in the restricted cohort, and for WT cases included in the restricted cohort is June 2021.

Supplementary Table 3. Number and types of *EZH2* mutations detected by Sanger sequencing of exons 16 and 18.

**Number of *EZH2* mutations detected using Sanger sequencing (*n* = 160)**	
**Exon 16**	
Y646N	14 (45.2%)
Y646F	7 (22.6%)
Y646S	5 (16.1%)
Y646H	3 (9.7%)
**Exon 18**	
A692V	1 (3.2%)
A682G	1 (3.2%)
**Total**	**31 (19.4%)**

Supplementary Table 4. Demographic and disease characteristics of patients in the restricted cohort (*n* = 29) as a function of *EZH2* status. The risk groups according to the Follicular Lymphoma International Prognostic Index (FLIPI) 1 and 2 are based on the number of risk factors: zero or one risk factor indicates low risk, two risk factors intermediate risk, and more than two risk factors high risk. Patients with bone marrow involvement were classified as having extranodal involvement. Bulk disease was defined as a tumor that was 7 cm or larger in the greatest dimension.

	***EZH2* WT**	***EZH2* mutant**
	**(*n* = 10)**	**(*n* = 19)**
**Age at diagnosis –yrs**		
Median	54.2	62.4
Range	35-75	37-84
**Sex –no. (%)**		
Female	7 (70)	13 (68)
Male	3 (30)	6 (32)
**Follow-up –yrs**		
Median	17	10
Range	8-24	1-21
**Death –no. (%)**	3 (30)	10 (53)
**Histological transformation –no. (%)**	3 (30)	3 (16)
**Ann Arbor stage at diagnosis –no. (%)**		
I	0	4 (21)
II	1 (10)	3 (16)
III	1 (10)	4 (21)
IV	7 (70)	8 (42)
Missing data	1 (10)	
**FLIPI1 risk status –no. (%)**		
Low	5 (50)	8 (42)
Intermediate	1 (10)	3 (16)
High	2 (20)	4 (21)
Missing data	2 (20)	4 (21)
**FLIPI2 risk status –no. (%)**		
Low	4 (40)	5 (26)
Intermediate	1 (10)	3 (16)
High	2 (20)	1 (5)
Missing data	3 (30)	10 (53)
**Bone marrow involvement –no./total no. (%)**	5/9 (56)	6/16 (38)
**Extranodal involvement –no./total no. (%)**	6/9 (67)	6/19 (32)
**Bulk disease –no./total no. (%)**	2/9 (22)	3/19 (16)

Supplementary Table 5. Previous history of breast cancer and treatments received among the patients in the restricted cohort (n = 29), in relation to *EZH2* status in FL and change in *EZH2* status during FL progression.

Patient/ sample ID	Sex	*EZH2* initial status	change of *EZH2* status over time	Prior breast cancer history	Treatment for breast cancer
P124	M	MUT	NO	NO	
P93	F	MUT	NO	NO	
P42	F	MUT	YES	NO	
P161	F	MUT	NO	YES	Surgery, radiotherapy
P12	F	MUT	NO	NO	
P23	M	MUT	NO	NO	
P25	F	MUT	NO	NO	
P29	F	MUT	NO	NO	
P54	M	MUT	NO	NO	
P70	M	MUT	NO	NO	
P89	F	MUT	NO	YES	Surgery, radiotherapy, hormonotherapy
P160	M	MUT	NO	NO	
P122	F	MUT	NO	NO	
P123	F	MUT	NO	NO	
P100	F	MUT	NO	NO	
P121	F	MUT	NO	NO	
P14	F	MUT	NO	NO	
P3	M	MUT	NO	NO	
P119	F	MUT	NO	YES	Chemotherapy, radiotherapy
P16	F	WT	YES	NO	
P28	F	WT	YES	YES	Surgery, radiotherapy, hormonotherapy
P162	F	WT	YES	NO	
P13	F	WT	NO	YES	Surgery, radiotherapy
P59	F	WT	NO	NO	
P96	M	WT	NO	NO	
P102	M	WT	NO	NO	
P99	M	WT	NO	NO	
P141	F	WT	NO	NO	
P20	F	WT	NO	NO	

Supplementary Table 6. Variant allelic frequency (VAF) of *EZH2* mutations in patient samples analyzed by ChIP-seq and RNA-seq in Figs. 4 and 5 and Supplementary Figs. 4 and 5.

***EZH2*-mutant sample identifier**	**VAF (%)**
**Fig. 4 and Supplementary Fig. 4**	
P23	22.79
P25	30.18
P29	18.83
P70	27.07
P122	23.70
P123	30.35
P100	51.84
P121	21.54
**Fig. 5 and Supplementary Fig. 5**	
P93T1	11.17
P93T2	10.32
P14T1	11.03
P14T2	12.65
P54T1	34.14
P54T2	10.87
P124T1	7.36
P124T2	12.83 (3.1% Y646H, 9.73% Y646N)
P119T1	15.1
P119T2	11.37
P89T1	38.97
P89T2	11.8
P162T1	11.28
P162T2	10.17
P42T1	11.67
P28T2	9
P28T3	11.2
P16T2	7.63

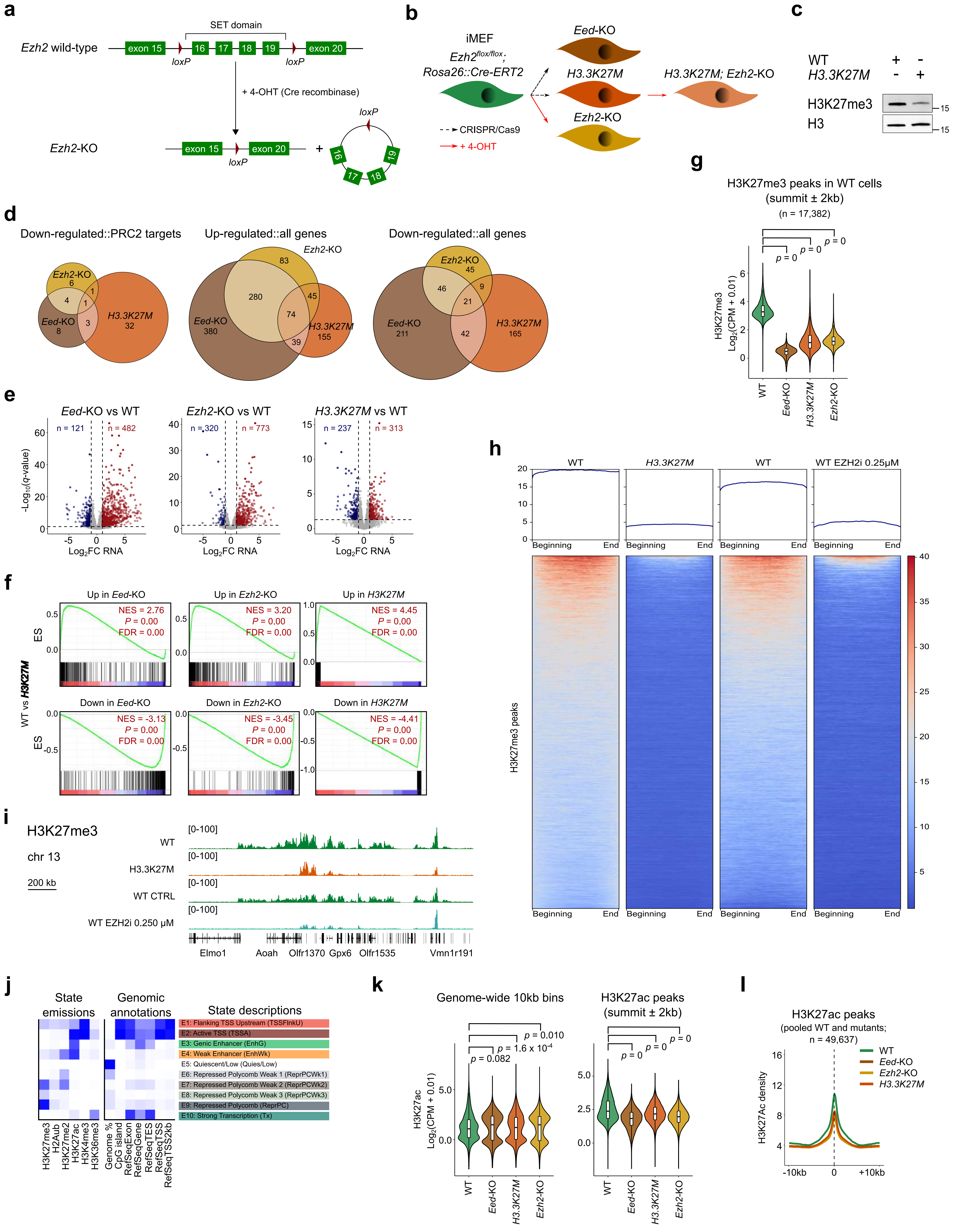

Supplementary Fig. 1. Oncogenic *Eed*-KO, *Ezh2*-KO and H3K27M mutations differ essentially in the degree of PRC2 loss of function they confer. **a** Schematic of *Ezh2*^{flox} allele used for conditional deletion of SET domain by Cre-ERT2 upon activation with 4-hydroxytamoxifen (4-OHT). **b** Construction of isogenic mutant iMEFs via CRISPR/Cas9-mediated gene editing and/or Cre-mediated recombination. **c** Western blot of H3K27me3 in iMEFs. Source data are provided as a Source Data file and at the end of the Supplementary Information file. The experiment was performed three times with similar results. **d** Overlap among differentially expressed genes identified by comparison of iMEFs of the indicated genotypes with WT, considering genes belonging to the categories shown above each diagram. **e** Comparative gene expression in the indicated pairs of iMEF lines, highlighting up-regulated (red) and down-regulated (blue) genes. Note the differing vertical axis scales. FC, fold change. **f** Pre-ranked gene-set enrichment analyses assessing the ranked enrichment of genes up- or down-regulated in *H3.3K27M* iMEFs (*versus* WT) among the sets of genes up- or down-regulated in the indicated mutant genotypes. **g** Violin and box plots showing normalized H3K27me3 CUT&RUN read counts for iMEFs within H3K27me3 peaks. *p*-value represents the result of an unpaired two-sided *t*-test. **h** Mean H3K27me3 density plots across the peaks analyzed in Fig. 1e, scaled to the same size, with density heatmaps of individual peaks shown below. EZH2i = EZH2 inhibitor UNC1999. **i** H3K27me3 CUT&RUN tracks for iMEFs across the same genomic window as Fig. 1d. **j** Heatmaps summarizing the 10 emission states of a ChromHMM model derived from CUT&RUN enrichment measurements of the indicated histone modifications in iMEFs, and showing the prevalence of each emission state at the indicated genomic annotations. **k** Violin and box plots showing normalized H3K27ac CUT&RUN read counts for iMEFs within all 10-kb bins genome-wide (left) or within H3K27ac peaks (right). *p*-values represent the result of unpaired two-sided *t*-tests. **l** Mean density plot of H3K27ac CUT&RUN reads in iMEFs across H3K27ac peaks.

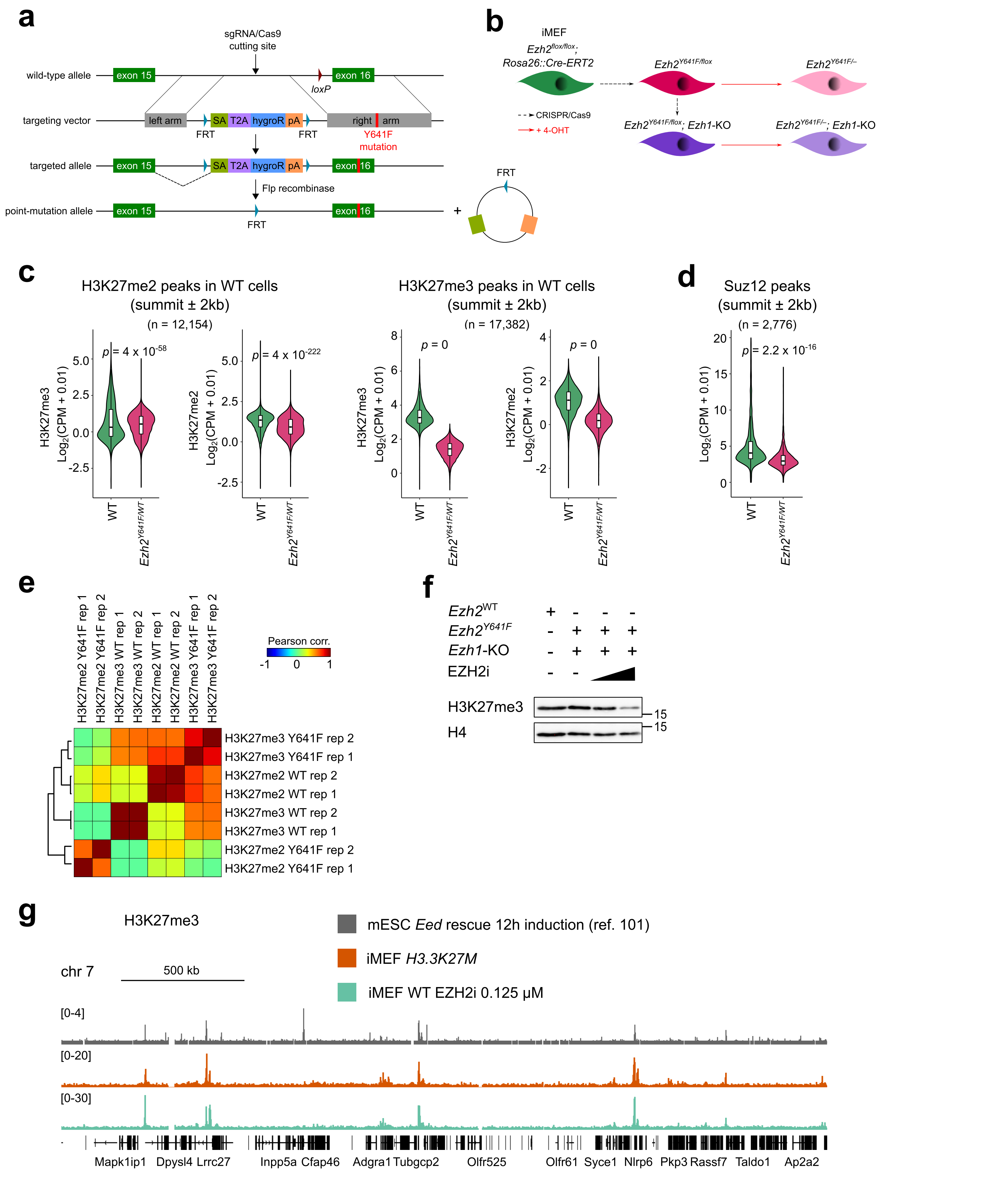

Supplementary Fig. 2. *Ezh2^{Y641F}* expression causes H3K27me3 to adopt a H3K27me2-like profile. a Schematic of gene editing strategy for introduction of the *Ezh2^{Y641F}* mutation, which coincides with insertion of a selectable marker and removal of a *loxP* site (see Supplementary Fig. 1a). Excision of the selectable marker through Flp-mediated recombination restores *Ezh2* gene function with the mutated allele. **b** Schematic illustrating the derivation of *Ezh2$^{Y641F/WT}$* immortalized mouse embryonic fibroblasts (iMEFs) and *Ezh1*-KO subclones via CRISPR/Cas9-mediated gene editing, and subsequent selective deletion of wild-type *Ezh2* via Cre-mediated recombination (see Supplementary Fig. 1a). **c** Violin and box plots showing normalized H3K27me3 and H3K27me2 CUT&RUN read counts for WT and *Ezh2$^{Y641F/WT}$* iMEFs within regions identified as H3K27me2 peaks in WT cells (left) or within regions identified as H3K27me3 peaks in WT cells (right). *p*-values represent the result of unpaired two-sided *t*-tests. **d** Violin and box plots showing normalized SUZ12 read counts for WT and *Ezh2$^{Y641F/WT}$* iMEFs within regions identified as SUZ12 peaks in WT cells. *p*-value represents the result of an unpaired two-sided *t*-test. **e** Correlation matrix of mapped reads in H3K27me3 and H3K27me2 CUT&RUN experiments conducted in duplicate in WT and *Ezh2$^{Y641F/WT}$* iMEFs. Samples are ordered by unsupervised hierarchical clustering. **f** Western blot analysis of the indicated proteins and post-translational modifications in isogenic immortalized mouse embryonic fibroblasts (iMEFs) of the indicated genotypes, either untreated (EZH2i-) or treated with the EZH2 inhibitor UNC1999 at 0.125 or 0.250 μM, as indicated by the height of the triangle. Source data are provided as a Source Data file and at the end of the Supplementary Information file. The experiment was performed three times with similar results. **g** H3K27me3 tracks from ChIP-seq in *Eed*-KO mouse embryonic stem cells (mESCs) 12h after re-expression of WT *Eed* (gray)[50] and from CUT&RUN in iMEFs of the indicated genotypes and conditions (orange and green), with corresponding genomic annotations shown below.

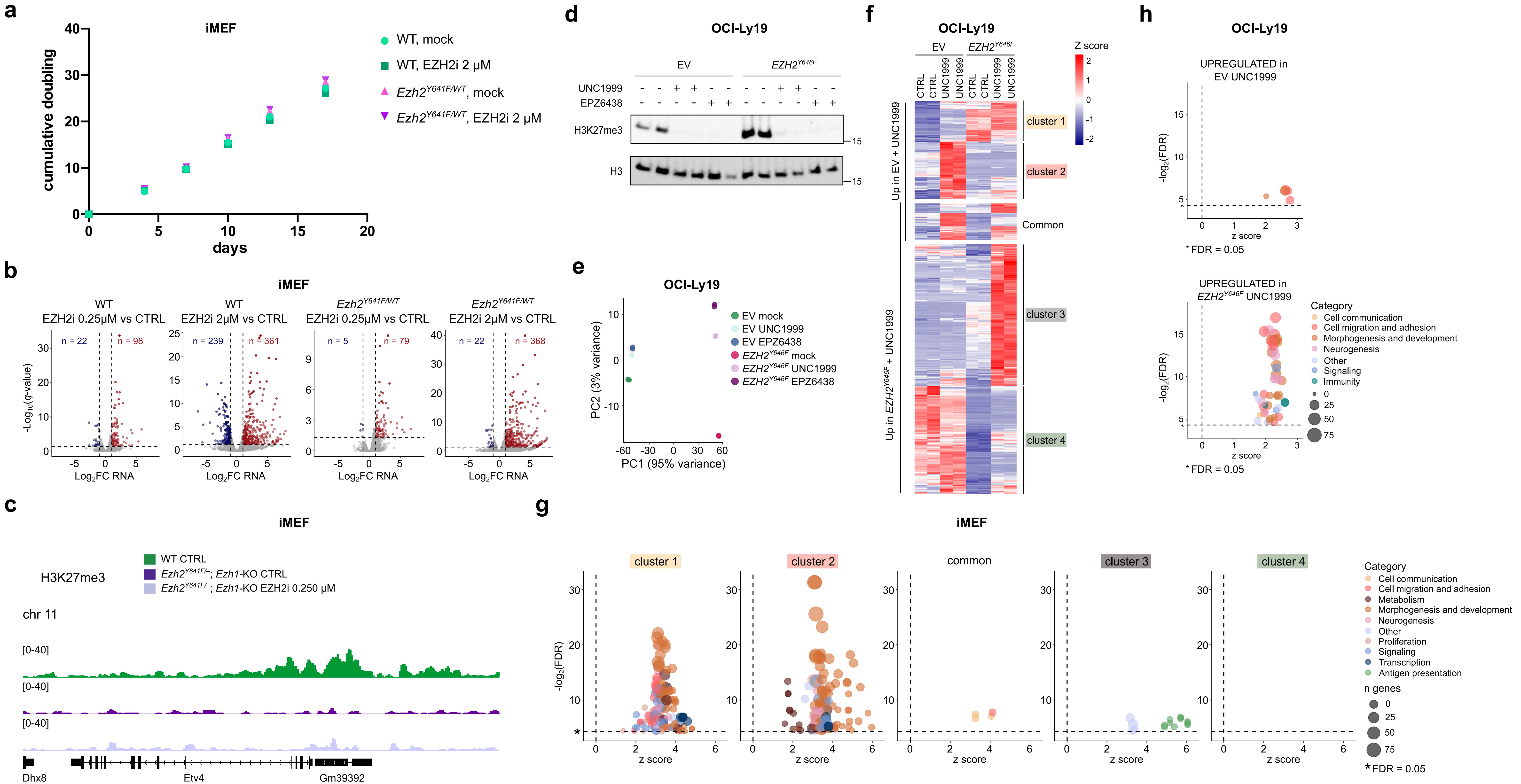

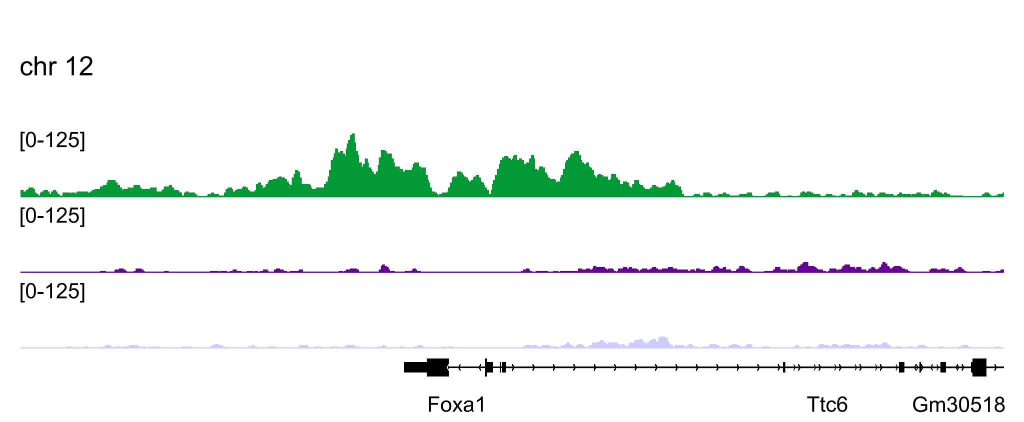

Supplementary Fig. 3. *Ezh2*^{Y641F} transforms the transcriptomic response to PRC2 inhibition. a Cumulative population doublings of WT and *Ezh2*$^{Y641F/WT}$ iMEFs either mock-treated or treated with 2 μM of the EZH2 inhibitor UNC1999 (EZH2i) for the indicated time. Source data are provided as a Source Data file. **b** Comparative gene expression between WT or *Ezh2*$^{Y641F/WT}$ iMEFs treated with the indicated doses of the EZH2 inhibitor UNC1999 and untreated cells of the same genotype (CTRL). Differentially expressed genes are highlighted in red (up-regulated) and blue (down-regulated). FC, fold change. **c** H3K27me3 CUT&RUN tracks for WT and *Ezh2*$^{Y641F/–}$; *Ezh1*-KO iMEFs, either untreated (CTRL) or treated with 0.25 μM of the EZH2 inhibitor UNC1999, with corresponding genomic annotations shown below. **d** Western blot analysis of H3K27me3 levels in OCI-Ly19 carrying either an empty vector (EV) or a vector for overexpression of *EZH2*^{Y646F} either untreated or treated with either of the EZH2 inhibitors UNC1999 (2 μM) or EPZ6438 (i.e., tazemetostat, 3 μM). Source data are provided as a Source Data file and at the end of the Supplementary Information file. The experiment was performed three times with similar results. **e** PCA of variance among RNA-seq experiments performed in duplicate on OCI-Ly19 cells carrying either an EV or an *EZH2*^{Y646F} overexpression vector and either mock-treated or treated with 2 μM UNC1999 or 3 μM EPZ6438. **f** Heatmap representing RNA-seq expression Z scores for all genes identified as up-regulated in either EV- or *EZH2*^{Y646F}-transduced OCI-Ly19 cells upon treatment with 2 μM UNC1999. Genes are ordered in clusters according to their genotype-specific up-regulation in response to 2 μM UNC1999 treatment and according to their expression level in the genotype in which they do not respond to UNC1999 treatment. **g** Bubble plots showing GO terms significantly enriched within each of the clusters of genes identified on the basis of their respective expression and up-regulation upon treatment with 2 μM UNC1999 in WT and *Ezh2*^{Y641F} iMEFs (see main Fig. 3e). GO, gene ontology. **h** Bubble plots showing GO terms significantly enriched in the sets of genes identified as up-regulated in either EV- or *EZH2*^{Y646F}-transduced OCI-Ly19 cells upon treatment with 2 μM UNC1999.

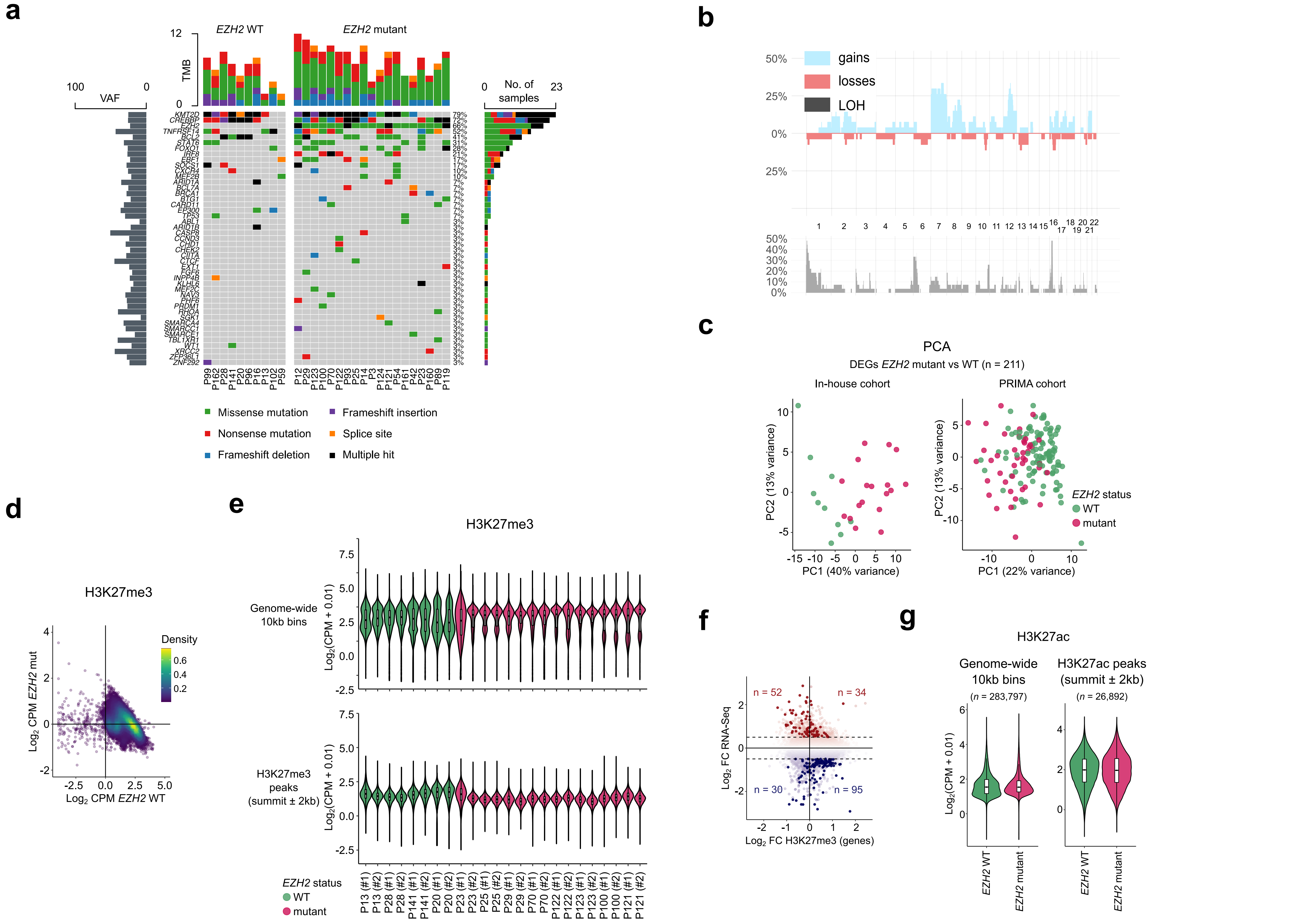

Supplementary Fig. 4. Aberrant H3K27me3 patterns are a consistent feature of *EZH2*-mutant follicular lymphomas. a Frequency and type of mutations affecting 45 genes identified in the restricted cohort defined in Fig. 4a. Rows represent genes and columns represent patient samples. For each patient, bars above show the number of genes containing a mutation. For each gene, bars to the right show the number of patients carrying a mutation and bars to the left show the mean variant allele frequency (VAF) among patients with mutations. **b** Summary of copy number variation analysis conducted on the restricted cohort. LOH, loss of heterozygosity. **c** Left, Principal component analysis (PCA) of variance among RNA-seq experiments performed on *EZH2*-WT and *EZH2*-mutant lymphoma samples, considering only genes identified as differentially expressed as a function of *EZH2* status (DEGs) as shown in Fig. 4c. Right, PCA of variance among RNA-seq samples from the Primary RItuximab and MAintenance (PRIMA) cohort[57], considering only genes identified as DEGs in our cohort. **d** MA plot of H3K27me3 ChIP-seq read counts comparing *EZH2*-mutant lymphoma samples and *EZH2*-WT lymphoma samples. **e** Violin and box plots showing normalized H3K27me3 ChIP-seq read counts for individual *EZH2*-WT and -mutant lymphoma samples, assayed in duplicate, within all bins of 10 kb across the genome (top) or within regions identified as enriched in H3K27me3 (bottom). **f** Scatterplot showing fold changes in RNA-seq gene expression values between *EZH2*-WT and -mutant lymphomas versus fold changes in H3K27me3 ChIP-seq read counts over corresponding gene bodies, with genes identified as differentially expressed highlighted in red (up-regulated) and blue (down-regulated). **g** Violin and box plots showing mean normalized H3K27ac ChIP-seq read counts for *EZH2*-WT and -mutant lymphoma samples within all bins of 10 kb across the genome (n = 283,797) (left) or within regions identified as H3K27ac peaks (n = 26,892) (right). Boxes represent median and first and third quartiles, while whiskers show minimum and maximum.

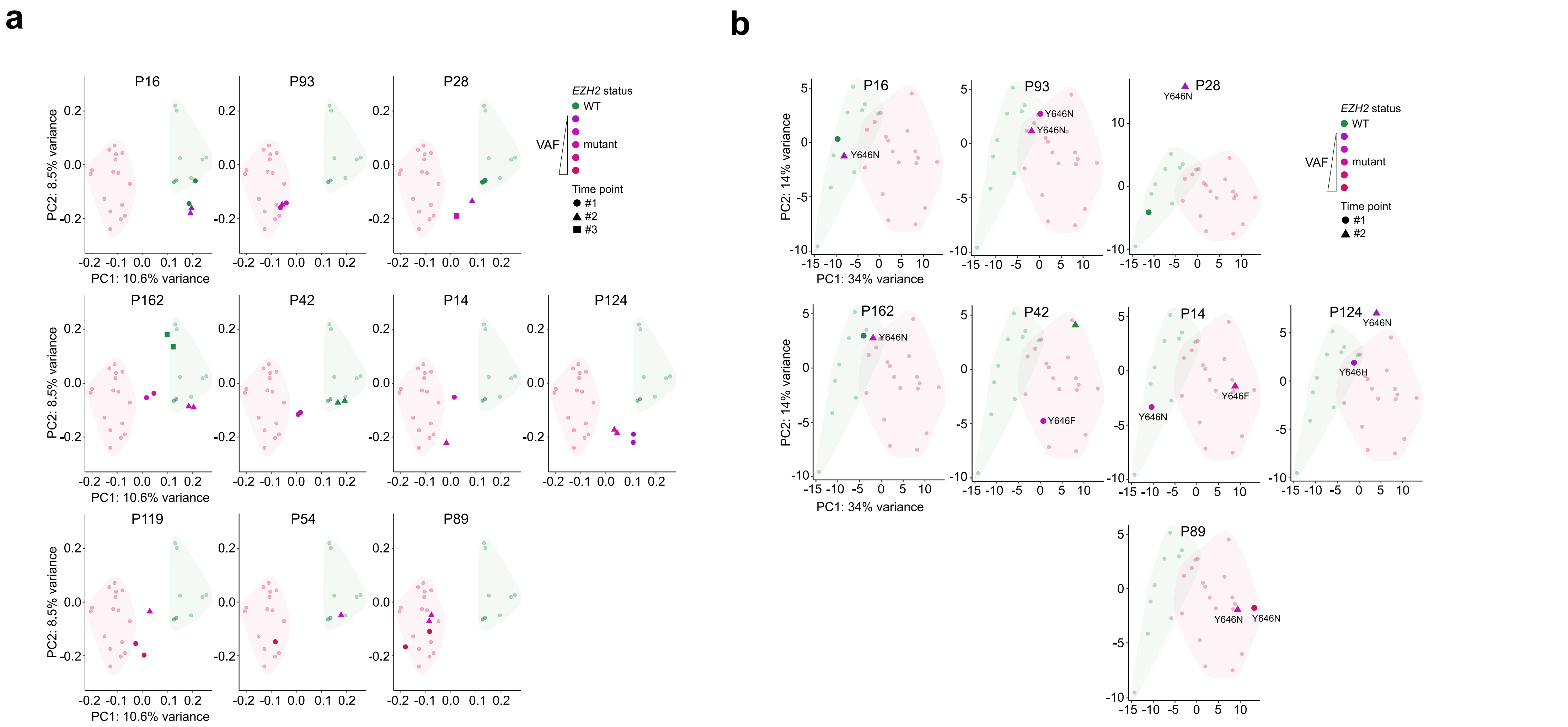

Supplementary Fig. 5. Changes in H3K27me3 distribution in follicular lymphoma track closely with changes in *EZH2* mutation status over time. a Principal component analysis (PCA) of variance among the full set of H3K27me3 ChIP-seq experiments performed on follicular lymphoma (FL) samples, considering only the 5000 most variable H3K27me3-enriched regions, as shown in Fig. 4g, with samples from 10 individual patients at distinct time points (see Fig. 4b) mapped onto the PCA shown in separate plots for each patient. *EZH2*-mutant data are color-coded according to variant allele frequency (VAF) as indicated. **b** PCA of variance among the full set of RNA-seq experiments performed on FL samples, considering only 211 differentially expressed genes displayed in Fig. 4c, with samples from 8 individual patients at distinct time points (see Fig. 4b) mapped onto the PCA shown in separate plots for each patient. *EZH2*-mutant data are color-coded according to VAF as indicated.

Source data for Supplementary Figure 1c

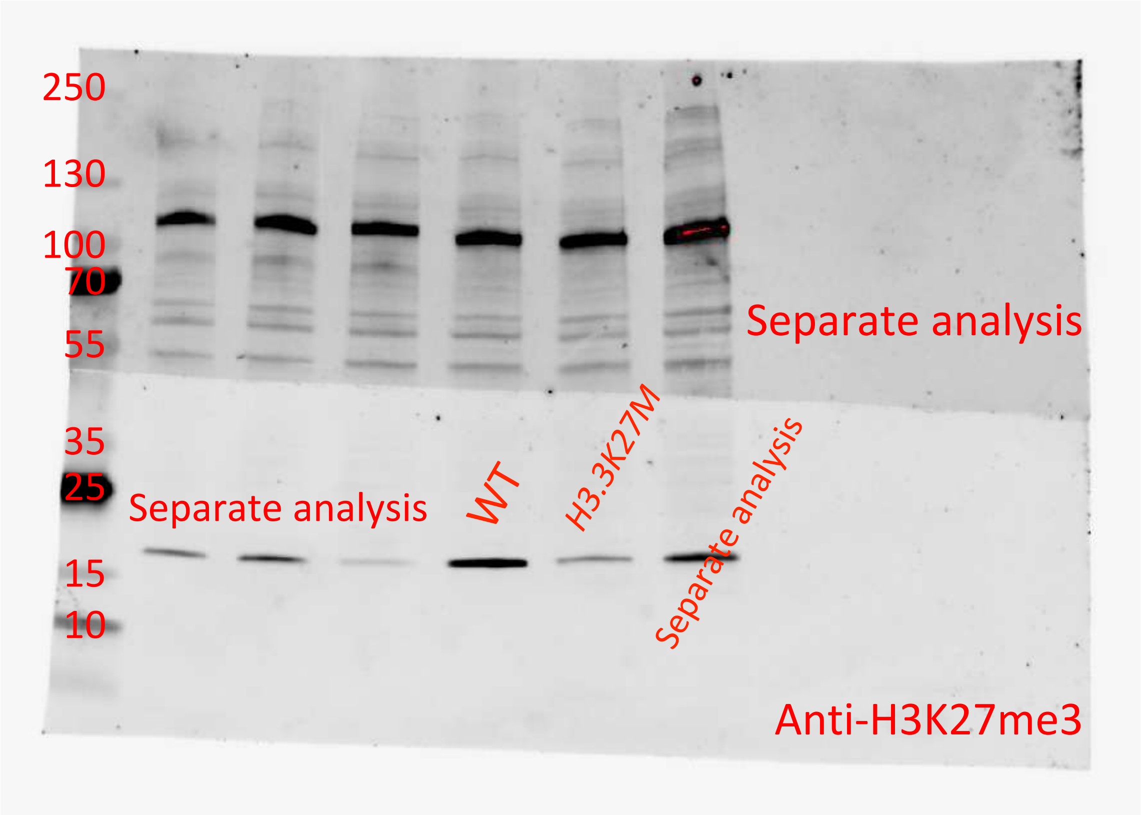

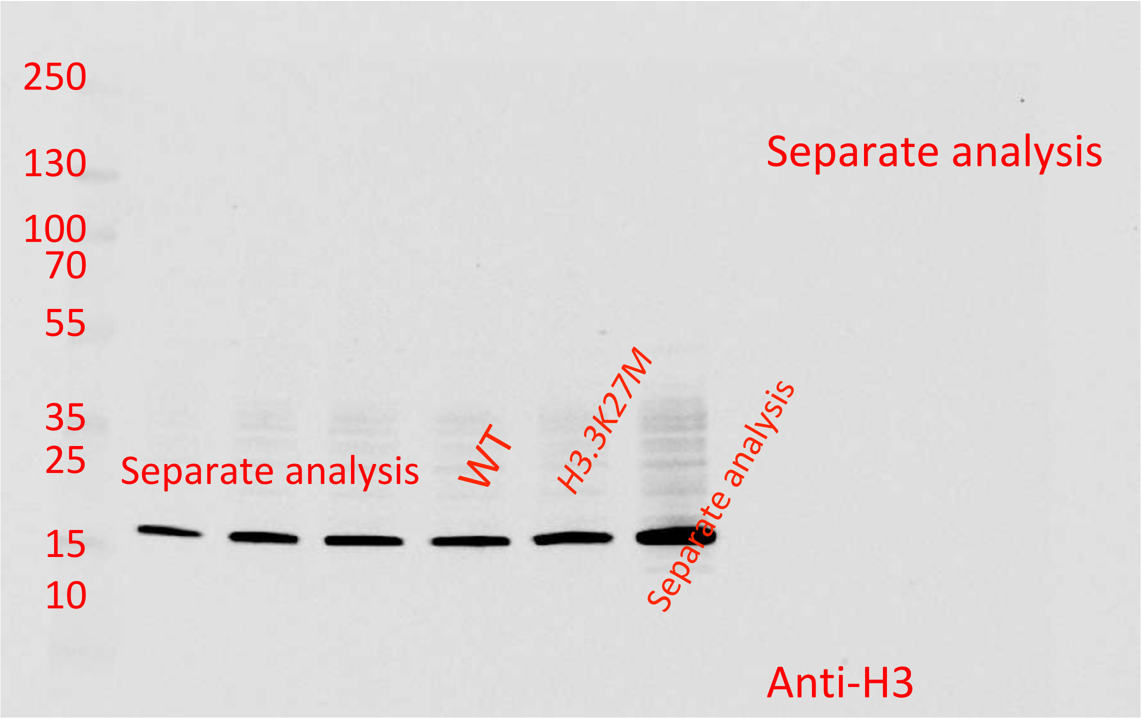

Source data for Supplementary Figure 2f

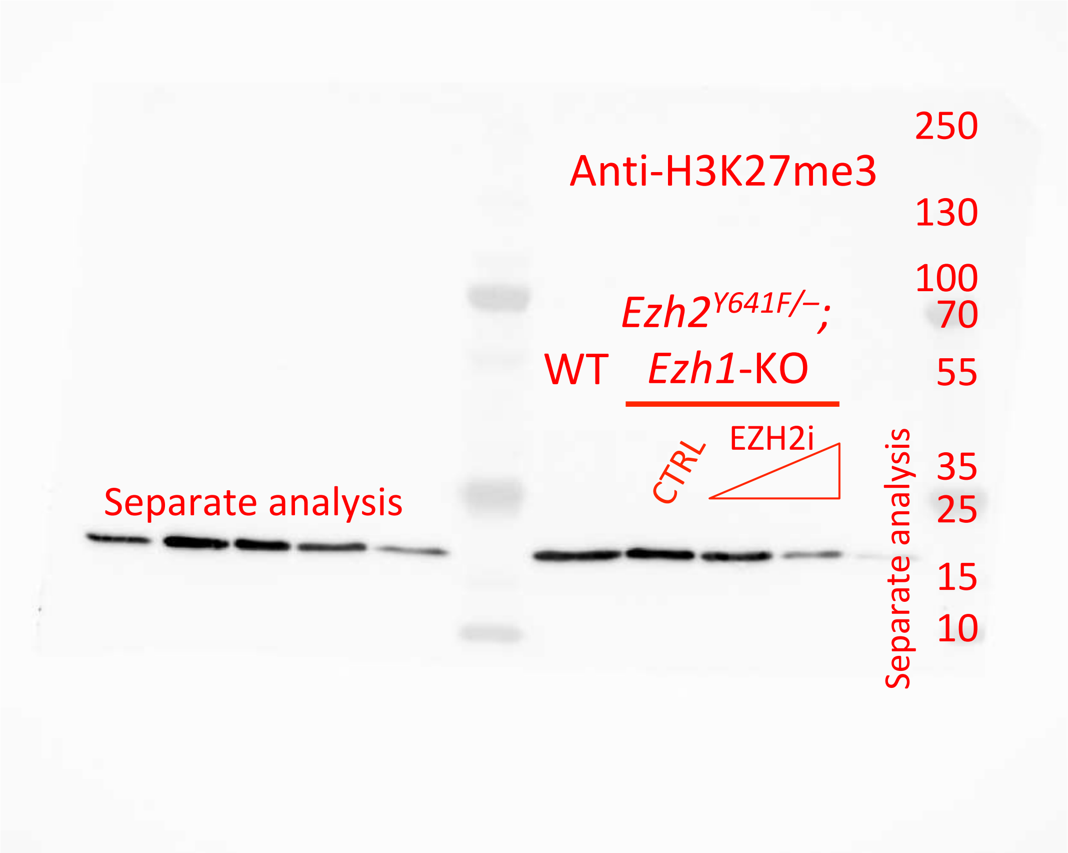

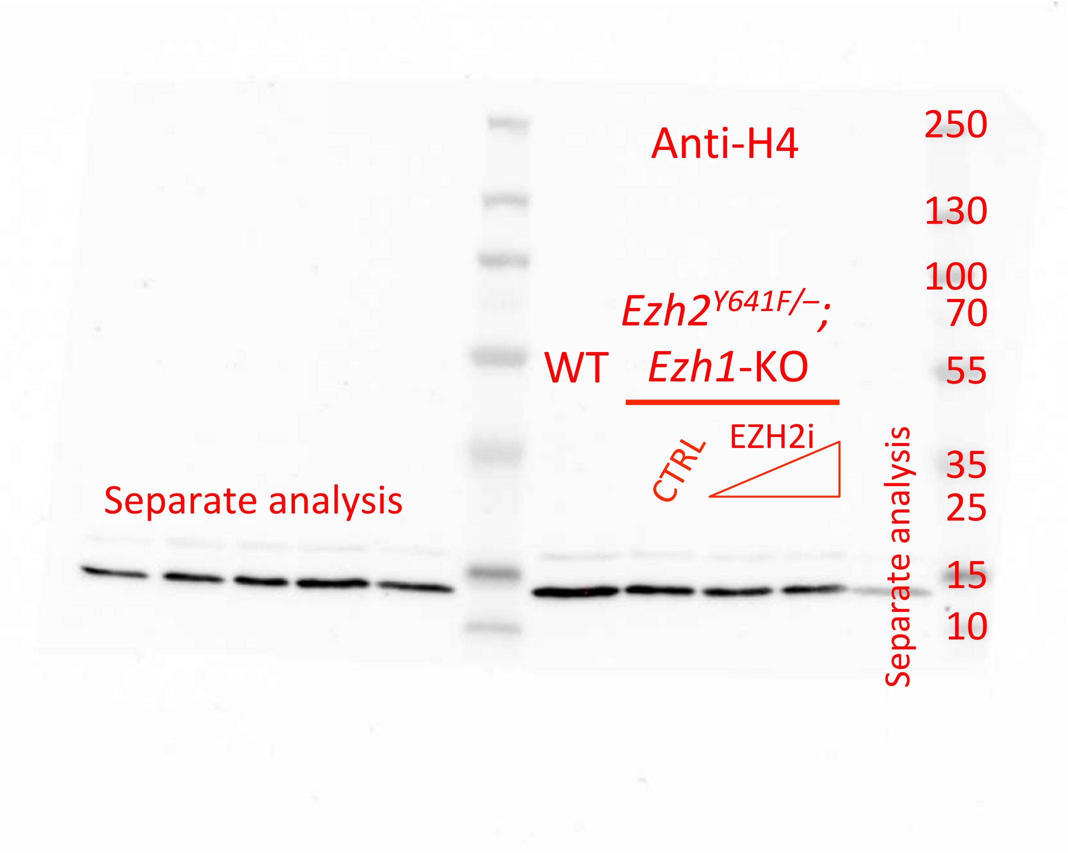

Source data for Supplementary Figure 3c

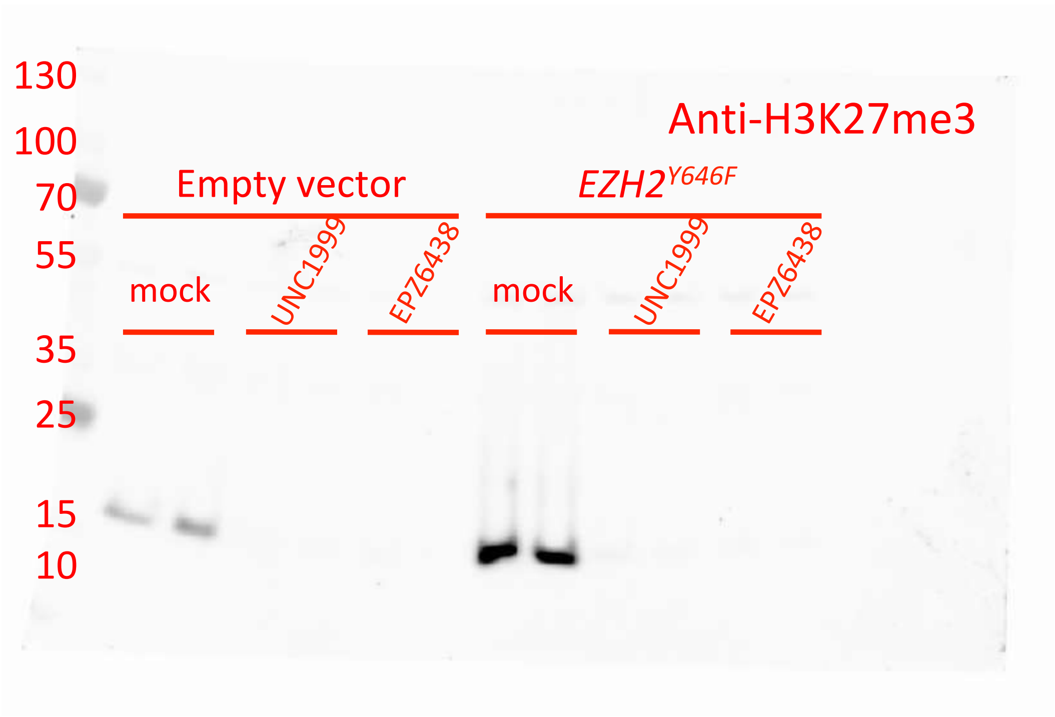

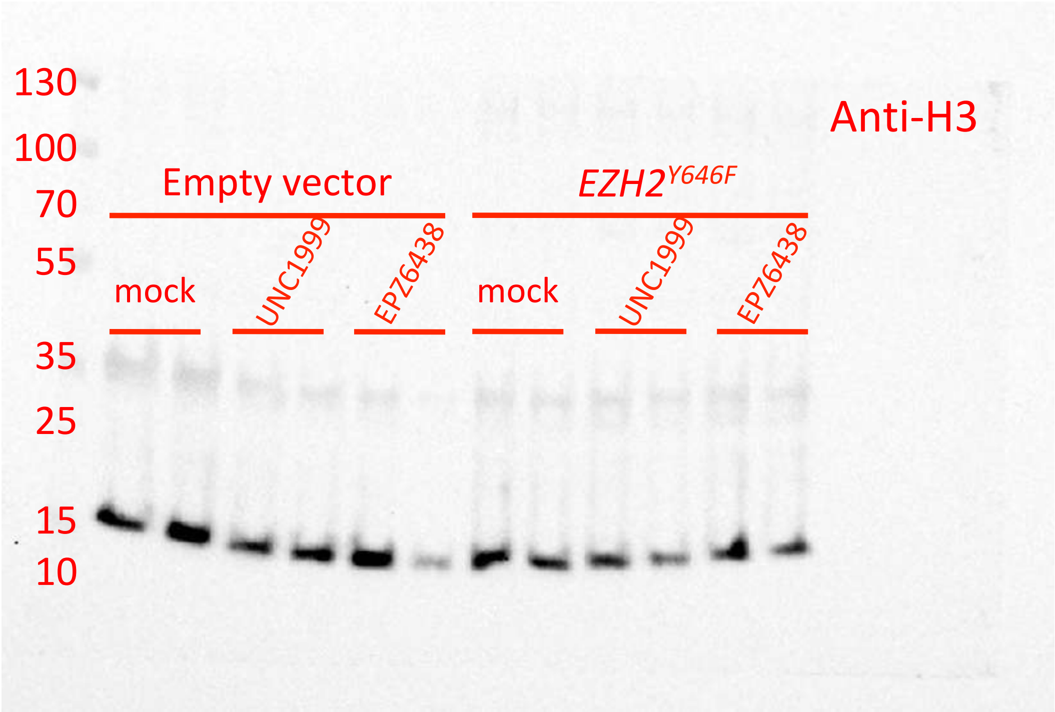

Supplement: Supplementary file 1 — Supplementary Information [file 41467_2024_47701_MOESM1_ESM.pdf]
