# Supplementary material for: EZH2 mutations in follicular lymphoma distort H3K27me3 profiles and alter transcriptional responses to PRC2 inhibition
Source: Nat Commun. 2024 Apr 24;15:3452. doi: 10.1038/s41467-024-47701-x (PMC11043461; doi:10.1038/s41467-024-47701-x)
Supplement: Supplementary file 3 — Description of Additional Supplementary Files [file 41467_2024_47701_MOESM3_ESM.pdf]

Description of Additional Supplementary Files

Supplementary Data 1. Microsoft Excel file: List of gene ontology terms significantly up-regulated in iMEFs treated with 2 µM of the EZH2 inhibitor UNC1999.

Supplementary Data 2. Microsoft Excel file: Genetic characterization using Determination of 571 Relevant Altered Genes in Oncology by NGS (DRAGON).

Supplementary Data 3. Microsoft Excel file: Oligonucleotides used for cloning single-guide-RNA expression constructs for CRISPR-mediated gene editing.
