# Supplementary material for: EZH2 mutations in follicular lymphoma distort H3K27me3 profiles and alter transcriptional responses to PRC2 inhibition
Source: Nat Commun. 2024 Apr 24;15:3452. doi: 10.1038/s41467-024-47701-x (PMC11043461; doi:10.1038/s41467-024-47701-x)
Supplement: Supplementary file 7 — Reporting Summary [file 41467_2024_47701_MOESM7_ESM.pdf]

nature portfolio

Corresponding author(s): Raphaël Margueron

Last updated by author(s): Mar 7, 2024

Reporting Summary

Nature Portfolio wishes to improve the reproducibility of the work that we publish. This form provides structure for consistency and transparency in reporting. For further information on Nature Portfolio policies, see our Editorial Policies and the Editorial Policy Checklist.

Statistics

For all statistical analyses, confirm that the following items are present in the figure legend, table legend, main text, or Methods section.

n/a	Confirmed	
☐	☒	The exact sample size (*n*) for each experimental group/condition, given as a discrete number and unit of measurement
☐	☒	A statement on whether measurements were taken from distinct samples or whether the same sample was measured repeatedly
☐	☒	The statistical test(s) used AND whether they are one- or two-sided<br>*Only common tests should be described solely by name; describe more complex techniques in the Methods section.*
☒	☐	A description of all covariates tested
☒	☐	A description of any assumptions or corrections, such as tests of normality and adjustment for multiple comparisons
☐	☒	A full description of the statistical parameters including central tendency (e.g. means) or other basic estimates (e.g. regression coefficient) AND variation (e.g. standard deviation) or associated estimates of uncertainty (e.g. confidence intervals)
☐	☒	For null hypothesis testing, the test statistic (e.g. *F*, *t*, *r*) with confidence intervals, effect sizes, degrees of freedom and *P* value noted<br>*Give P values as exact values whenever suitable.*
☒	☐	For Bayesian analysis, information on the choice of priors and Markov chain Monte Carlo settings
☒	☐	For hierarchical and complex designs, identification of the appropriate level for tests and full reporting of outcomes
☒	☐	Estimates of effect sizes (e.g. Cohen's *d*, Pearson's *r*), indicating how they were calculated

Our web collection on statistics for biologists contains articles on many of the points above.

Software and code

Policy information about availability of computer code

Data collection	Western blots were imaged using the standard software available with the ChemiDoc System (Biorad).
Data analysis	The Methods section details all the open-source software tools that were used to process the next-generation sequencing data analyzed in the study. They are as follows:<br>TrimGalore (v0.4.0)<br>Cutadapt (v1.8.2)<br>STAR (v2.7.0a) aligner<br>FeatureCounts (Subread v1.5.1)<br>limma (v3.58.1) (BioconductoR)<br>DESeq2 (v1.42.0) (BioconductoR)<br>ggplot2 (v3.5.0) (CRAN)<br>Bowtie2 (v2.2.5)<br>Picard Tools MarkDuplicates (v1.97)<br>DeepTools (v3.0.2)<br>csaw (v1.36.1) (BioconductoR)<br>UCSC tools (v2017.05.03)<br>MACS2 (v2.0.10)<br>BEDtools merge (v2.2.2)<br>ChromHMM (v1.25)<br>MEME AME (v5.5.5)<br>STRING (v11.5)

Cytoscape (v3.10.1)
GSEA_MacApp_4.3.3
Varscan2 (v2.4.3)
Facets (v0.5.1)

For manuscripts utilizing custom algorithms or software that are central to the research but not yet described in published literature, software must be made available to editors and reviewers. We strongly encourage code deposition in a community repository (e.g. GitHub). See the Nature Portfolio guidelines for submitting code & software for further information.

Data

Policy information about availability of data

All manuscripts must include a data availability statement. This statement should provide the following information, where applicable:

- Accession codes, unique identifiers, or web links for publicly available datasets
- A description of any restrictions on data availability
- For clinical datasets or third party data, please ensure that the statement adheres to our policy

RNA-seq, CUT&RUN-seq and ChIP-seq data for this study have been deposited in the Gene Expression Omnibus under accession no. GSE218717, which is publicly available as of March 6, 2024.
The following databases and datasets were used for data analysis in this study:
Mouse reference genome mm10
Mouse genome annotation GENCODE: release 23/GRCm38.p6
GSM2475229 [https://www.ncbi.nlm.nih.gov/geo/query/acc.cgi?acc=GSM2475229]
http://geneontology.org/
Drosophila melanogaster reference genome dm6
PRIMA trial (NCT00140582) RNA-seq raw data (courtesy of Drs. Huet, Tesson and Salles (Hospices Civils de Lyon, Pierre-Bénite; INSERM U1052, Université de Lyon; Carnot Calym, Pierre-Bénite; France)
Human reference genome hg19
COSMIC Cancer Gene Census (CGC) v86

Research involving human participants, their data, or biological material

Policy information about studies with human participants or human data. See also policy information about sex, gender (identity/presentation), and sexual orientation and race, ethnicity and racism.

Reporting on sex and gender	The sex of the patients whose follicular lymphoma (FL) biopsies were analyzed in the study was determined from medical records and is reported in Supplementary Tables 4, 6 and 7. Patients were not selected on the basis of sex; the greater number of female patients in our cohort is the consequence of individuals being referred for FL after having received treatment for breast cancer at our institution. Given that the numbers of EZH2-wild-type and EZH2-mutant patients of each sex are modest, we refrain from any conclusions regarding the effect of sex on our observations.
Reporting on race, ethnicity, or other socially relevant groupings	No socially relevant groupings were considered in the design of our study.
Population characteristics	The covariate-relevant characteristics of the patients whose follicular (FL) biopsies were analyzed in the study are reported in Supplementary Tables 3 through 9.
Recruitment	All patients with follicular lymphoma (FL) were recruited through Institut Curie (Paris, France) based on frozen sample availability and clinical history at the Biological Resource Center. In total, 160 patients were diagnosed and/or treated at Institut Curie for grade 1/2/3a FL between 1988 and 2017.
Ethics oversight	All tissue samples were fully anonymized before processing and sequencing. Study approval was first provided by the institutional review board and local ethical committee (Groupe Thématique de Travail – Hematology section, Institut Curie), under project ID BS#2014-311, when written informed patient consent was available, or else by the Comité de Protection des Personnes Sud Méditerrannée I, under project ID#RCB2019-A000620-57, when written consent was not available due to death or lost to follow-up.

Note that full information on the approval of the study protocol must also be provided in the manuscript.

Field-specific reporting

Please select the one below that is the best fit for your research. If you are not sure, read the appropriate sections before making your selection.

☒ Life sciences ☐ Behavioural & social sciences ☐ Ecological, evolutionary & environmental sciences

For a reference copy of the document with all sections, see nature.com/documents/nr-reporting-summary-flat.pdf

Life sciences study design

All studies must disclose on these points even when the disclosure is negative.

Sample size	The number of patients whose follicular (FL) biopsies were analyzed in the study was determined based on frozen sample availability and clinical history at the Biological Resource Center. The size of the sample chosen for in-depth analysis was decided on the basis of available material.<br>For each cell-culture-based experiment, two independent biological replicates were conducted, or three for readouts not requiring next-generation sequencing, following common practices in the field.
Data exclusions	No data were excluded from the analyses.
Replication	Replication of cell-culture-based assays was performed by including multiple biological replicates, as described in "Sample size" above. All attempts at replication were successful.<br>Replication of patient-sample-based assays was attempted by comparing to data from the independent PRIMA cohort, as detailed in Fig. 4d and Supplementary Fig. 4c of the manuscript.
Randomization	Randomization was not relevant to our study because we did not need to assign a fixed number of samples or individuals to mutually exclusive experimental treatments.
Blinding	Blinding was not relevant to our study because only objective measurements were made.

Reporting for specific materials, systems and methods

We require information from authors about some types of materials, experimental systems and methods used in many studies. Here, indicate whether each material, system or method listed is relevant to your study. If you are not sure if a list item applies to your research, read the appropriate section before selecting a response.

Materials & experimental systems

n/a	Involved in the study
☐	☒ Antibodies
☐	☒ Eukaryotic cell lines
☒	☐ Palaeontology and archaeology
☒	☐ Animals and other organisms
☒	☐ Clinical data
☒	☐ Dual use research of concern
☒	☐ Plants

Methods

n/a	Involved in the study
☐	☒ ChIP-seq
☒	☐ Flow cytometry
☒	☐ MRI-based neuroimaging

Antibodies

Antibodies used

Antibody Host Source Clone/identifier
EZH2 rabbit polyclonal homemade (ref. 33)
H3K27me3 rabbit monoclonal CST C36B11
H3K27me2 mouse monoclonal Active Motif 324
H3K27me1 mouse monoclonal Active Motif 321
EED rabbit polyclonal homemade (ref. 33)
H3.3K27M rabbit monoclonal Millipore RM192
H2Aub rabbit monoclonal CST 8240S
H3K27ac rabbit polyclonal Abcam Ab4729
H3K4me3 rabbit monoclonal CST C42D8
H3K36me3 rabbit polyclonal Abcam Ab9050
H4 rabbit polyclonal Active Motif AB_2636967
SUZ12 rabbit monoclonal CST D39F6
Rabbit IgG goat polyclonal WB secondary BioRad 12004161
Mouse IgG goat polyclonal WB secondary BioRad STAR117D800GA

Validation

EZH2: disappearance of band in Western blot upon genetic knockout of Ezh2 (Fig. 1a)
H3K27me3, H3K27me2, H3K27me1, EED: disappearance of bands in Western blot upon genetic knockout of Eed (Fig. 1a)
H3.3K27M: appearance of specific band in Western blot upon introduction of H3.3K27M mutation by gene editing (Fig. 1a)
H2Aub: disappearance of band in Western blot upon genetic knockout of RING1A/B (PMID 30664650, Fig. 6b)
H3K27ac: specific binding of the antibody to H3K27ac peptides and not unmodified H3 peptides or other acetylated H3 or H4 peptides, according to the manufacturer: https://www.abcam.com/products/primary-antibodies/histone-h3-acetyl-k27-antibody-chip-grade-ab4729.html#lb
H3K4me3: the manufacturer reports that "Tri-Methyl-Histone H3 (Lys4) Antibody detects endogenous levels of histone H3 when tri-methylated on Lys4. This antibody shows some cross-reactivity with histone H3 that is di-methylated on Lys4, but does not cross-react with non-methylated or mono-methylated histone H3 Lys4." (https://www.cellsignal.com/products/primary-antibodies/tri-methyl-histone-h3-lys4-c42d8-rabbit-mab/9751)

H3K36me3: strong reduction in the intensity of band in Western blot upon genetic knockout of Setd2 (PMID 34782763, Extended Data Fig. 9a)
H4: the manufacturer reports: "Detection of Histone H4 by Western blot. The analysis was performed using HeLa acid extract (20 µg) and probed with Histone H4 pAb at a 1:1,000 dilution." (https://www.activemotif.com/documents/tds/39269.pdf)
SUZ12: the manufacturer reports high enrichment of Hoxa loci in a chromatin immunoprecipitation conducted on NCCIT cells using the antibody, with no concurrent enrichment of alpha-satellite sequences. (Image 7/11 in image gallery — https://www.cellsignal.com/products/primary-antibodies/suz12-d39f6-xp-rabbit-mab/3737)

Eukaryotic cell lines

Policy information about cell lines and Sex and Gender in Research

Cell line source(s)	Immortalized mouse embryonic fibroblasts (iMEFs) were obtained by isolating MEFs from a female Ezh2flox/flox;Rosa26::Cre-ERT2 13.5-d-old embryo, infecting them with pMXs-hc-MYC (Addgene, catalog no. 17220) and deriving a clone via limiting dilution, as described in PMID 34782763 (in which the cell line is referred to as "iMEF B").<br>OCI-Ly19 cells and their EZH2-Y646F-transduced counterparts, a kind gift from N. Katanayeva and E. Oricchio, were established from the DLBCL tumor of a female patient.
Authentication	Authentication of Ezh2flox/flox;Rosa26::Cre-ERT2 iMEFs was done by confirming loss of EZH2 protein by Western blot after treatment with 4-hydroxytamoxifen (Fig. 1a, Supplementary Fig. 1a, b).<br>Authentication of empty-vector- and EZH2-Y646F-transduced OCI-Ly19 cells was done by confirming increased H3K27me3 levels in EZH2-Y646F-transduced cells (Supplementary Fig. 3c).
Mycoplasma contamination	Cell lines were tested on a monthly basis for mycoplasma contamination by PCR and were consistently confirmed to be negative in an assay that included a positive control sample.
Commonly misidentified lines<br>(See ICLAC register)	No commonly misidentified cell lines were used in this study.

Plants

Seed stocks	Not applicable.
Novel plant genotypes	Not applicable.
Authentication	Not applicable.

Data deposition

☒ Confirm that both raw and final processed data have been deposited in a public database such as GEO.

☒ Confirm that you have deposited or provided access to graph files (e.g. BED files) for the called peaks.

Data access links<br>*May remain private before publication.*	CUT&RUN-seq and ChIP-seq data for this study have been deposited in the Gene Expression Omnibus under accession no. GSE218717. They can be accessed using the reviewer token anoxeceihfelbaf.
Files in database submission	Raw files:<br>D387C101_trimmed.R1.fastq.gz D387C101_trimmed.R2.fastq.gz<br>D387C102_trimmed.R1.fastq.gz D387C102_trimmed.R2.fastq.gz<br>D387C103_trimmed.R1.fastq.gz D387C103_trimmed.R2.fastq.gz<br>D387C104_trimmed.R1.fastq.gz D387C104_trimmed.R2.fastq.gz<br>D387C105_trimmed.R1.fastq.gz D387C105_trimmed.R2.fastq.gz<br>D387C106_trimmed.R1.fastq.gz D387C106_trimmed.R2.fastq.gz<br>D387C107_trimmed.R1.fastq.gz D387C107_trimmed.R2.fastq.gz<br>D387C108_trimmed.R1.fastq.gz D387C108_trimmed.R2.fastq.gz<br>D387C109_trimmed.R1.fastq.gz D387C109_trimmed.R2.fastq.gz<br>D387C110_trimmed.R1.fastq.gz D387C110_trimmed.R2.fastq.gz<br>D387C131_trimmed.R1.fastq.gz D387C131_trimmed.R2.fastq.gz<br>D387C132_trimmed.R1.fastq.gz D387C132_trimmed.R2.fastq.gz<br>D387C133_trimmed.R1.fastq.gz D387C133_trimmed.R2.fastq.gz<br>D387C134_trimmed.R1.fastq.gz D387C134_trimmed.R2.fastq.gz<br>D387C135_trimmed.R1.fastq.gz D387C135_trimmed.R2.fastq.gz<br>D387C136_trimmed.R1.fastq.gz D387C136_trimmed.R2.fastq.gz<br>D387C137_trimmed.R1.fastq.gz D387C137_trimmed.R2.fastq.gz<br>D387C138_trimmed.R1.fastq.gz D387C138_trimmed.R2.fastq.gz

D387C139_trimmed.R1.fastq.gz D387C139_trimmed.R2.fastq.gz
D387C140_trimmed.R1.fastq.gz D387C140_trimmed.R2.fastq.gz
D127C73.R1.fastq.gz D127C73.R2.fastq.gz
D127C74.R1.fastq.gz D127C74.R2.fastq.gz
D127C75.R1.fastq.gz D127C75.R2.fastq.gz
D127C76.R1.fastq.gz D127C76.R2.fastq.gz
D127C77.R1.fastq.gz D127C77.R2.fastq.gz
D127C78.R1.fastq.gz D127C78.R2.fastq.gz
D127C79.R1.fastq.gz D127C79.R2.fastq.gz
D127C80.R1.fastq.gz D127C80.R2.fastq.gz
D127C81.R1.fastq.gz D127C81.R2.fastq.gz
D127C82.R1.fastq.gz D127C82.R2.fastq.gz
D127C53.R1.fastq.gz D127C53.R2.fastq.gz
D127C54.R1.fastq.gz D127C54.R2.fastq.gz
D127C55.R1.fastq.gz D127C55.R2.fastq.gz
D127C56.R1.fastq.gz D127C56.R2.fastq.gz
D127C57.R1.fastq.gz D127C57.R2.fastq.gz
D127C58.R1.fastq.gz D127C58.R2.fastq.gz
D127C59.R1.fastq.gz D127C59.R2.fastq.gz
D127C60.R1.fastq.gz D127C60.R2.fastq.gz
D127C61.R1.fastq.gz D127C61.R2.fastq.gz
D127C62.R1.fastq.gz D127C62.R2.fastq.gz
D127C63.R1.fastq.gz D127C63.R2.fastq.gz
D127C64.R1.fastq.gz D127C64.R2.fastq.gz
D127C65.R1.fastq.gz D127C65.R2.fastq.gz
D127C66.R1.fastq.gz D127C66.R2.fastq.gz
D127C67.R1.fastq.gz D127C67.R2.fastq.gz
D127C68.R1.fastq.gz D127C68.R2.fastq.gz
D127C69.R1.fastq.gz D127C69.R2.fastq.gz
D127C70.R1.fastq.gz D127C70.R2.fastq.gz
D127C71.R1.fastq.gz D127C71.R2.fastq.gz
D127C72.R1.fastq.gz D127C72.R2.fastq.gz
D387C121_trimmed.R1.fastq.gz D387C121_trimmed.R2.fastq.gz
D387C122_trimmed.R1.fastq.gz D387C122_trimmed.R2.fastq.gz
D387C123_trimmed.R1.fastq.gz D387C123_trimmed.R2.fastq.gz
D387C124_trimmed.R1.fastq.gz D387C124_trimmed.R2.fastq.gz
D387C125_trimmed.R1.fastq.gz D387C125_trimmed.R2.fastq.gz
D387C126_trimmed.R1.fastq.gz D387C126_trimmed.R2.fastq.gz
D387C127_trimmed.R1.fastq.gz D387C127_trimmed.R2.fastq.gz
D387C128_trimmed.R1.fastq.gz D387C128_trimmed.R2.fastq.gz
D387C129_trimmed.R1.fastq.gz D387C129_trimmed.R2.fastq.gz
D387C130_trimmed.R1.fastq.gz D387C130_trimmed.R2.fastq.gz
D387C153_trimmed.R1.fastq.gz D387C153_trimmed.R2.fastq.gz
D387C154_trimmed.R1.fastq.gz D387C154_trimmed.R2.fastq.gz
D387C155_trimmed.R1.fastq.gz D387C155_trimmed.R2.fastq.gz
D387C156_trimmed.R1.fastq.gz D387C156_trimmed.R2.fastq.gz
D387C157_trimmed.R1.fastq.gz D387C157_trimmed.R2.fastq.gz
D387C158_trimmed.R1.fastq.gz D387C158_trimmed.R2.fastq.gz
D387C160_trimmed.R1.fastq.gz D387C160_trimmed.R2.fastq.gz
D387C161_trimmed.R1.fastq.gz D387C161_trimmed.R2.fastq.gz
D387C162_trimmed.R1.fastq.gz D387C162_trimmed.R2.fastq.gz
D387C163_trimmed.R1.fastq.gz D387C163_trimmed.R2.fastq.gz
D387C164_trimmed.R1.fastq.gz D387C164_trimmed.R2.fastq.gz
D387C165_trimmed.R1.fastq.gz D387C165_trimmed.R2.fastq.gz
D233C05.R1.fastq.gz D233C05.R2.fastq.gz
D233C06.R1.fastq.gz D233C06.R2.fastq.gz
D256-D251C26.R1.fastq.gz D256-D251C26.R2.fastq.gz
D256-D251C27.R1.fastq.gz D256-D251C27.R2.fastq.gz
D256-D251C29.R1.fastq.gz D256-D251C29.R2.fastq.gz
D256-D251C30.R1.fastq.gz D256-D251C30.R2.fastq.gz
D233C14.R1.fastq.gz D233C14.R2.fastq.gz
D233C15.R1.fastq.gz D233C15.R2.fastq.gz
D233C17.R1.fastq.gz D233C17.R2.fastq.gz
D233C18.R1.fastq.gz D233C18.R2.fastq.gz
D233C20.R1.fastq.gz D233C20.R2.fastq.gz
D233C21.R1.fastq.gz D233C21.R2.fastq.gz
D233C26.R1.fastq.gz D233C26.R2.fastq.gz
D233C27.R1.fastq.gz D233C27.R2.fastq.gz
D256-D251C11.R1.fastq.gz D256-D251C11.R2.fastq.gz
D256-D251C12.R1.fastq.gz D256-D251C12.R2.fastq.gz
D256-D251C17.R1.fastq.gz D256-D251C17.R2.fastq.gz
D256-D251C18.R1.fastq.gz D256-D251C18.R2.fastq.gz
D256-D251C20.R1.fastq.gz D256-D251C20.R2.fastq.gz
D256-D251C21.R1.fastq.gz D256-D251C21.R2.fastq.gz
D256-D251C23.R1.fastq.gz D256-D251C23.R2.fastq.gz
D256-D251C24.R1.fastq.gz D256-D251C24.R2.fastq.gz

D164C07.R1.fastq.gz D164C07.R2.fastq.gz
D164C09.R1.fastq.gz D164C09.R2.fastq.gz
D330C05_trimmed.R1.fastq.gz D330C05_trimmed.R2.fastq.gz
D330C06_trimmed.R1.fastq.gz D330C06_trimmed.R2.fastq.gz
D233C23.R1.fastq.gz D233C23.R2.fastq.gz
D330C07_trimmed.R1.fastq.gz D330C07_trimmed.R2.fastq.gz
D233C08.R1.fastq.gz D233C08.R2.fastq.gz
D233C09.R1.fastq.gz D233C09.R2.fastq.gz
D330C12_trimmed.R1.fastq.gz D330C12_trimmed.R2.fastq.gz
D330C13_trimmed.R1.fastq.gz D330C13_trimmed.R2.fastq.gz
D330C15_trimmed.R1.fastq.gz D330C15_trimmed.R2.fastq.gz
D330C16_trimmed.R1.fastq.gz D330C16_trimmed.R2.fastq.gz
D330C17_trimmed.R1.fastq.gz D330C17_trimmed.R2.fastq.gz
D330C18_trimmed.R1.fastq.gz D330C18_trimmed.R2.fastq.gz
D164C17.R1.fastq.gz D164C17.R2.fastq.gz
D164C19.R1.fastq.gz D164C19.R2.fastq.gz
D256-D251C02.R1.fastq.gz D256-D251C02.R2.fastq.gz
D256-D251C03.R1.fastq.gz D256-D251C03.R2.fastq.gz
D330C19_trimmed.R1.fastq.gz D330C19_trimmed.R2.fastq.gz
D330C20_trimmed.R1.fastq.gz D330C20_trimmed.R2.fastq.gz
D330C21_trimmed.R1.fastq.gz D330C21_trimmed.R2.fastq.gz
D347-D340C02_trimmed.R1.fastq.gz D347-D340C02_trimmed.R2.fastq.gz
D347-D340C03_trimmed.R1.fastq.gz D347-D340C03_trimmed.R2.fastq.gz
D347-D340C05_trimmed.R1.fastq.gz D347-D340C05_trimmed.R2.fastq.gz
D347-D340C07_trimmed.R1.fastq.gz D347-D340C07_trimmed.R2.fastq.gz
D347-D340C08_trimmed.R1.fastq.gz D347-D340C08_trimmed.R2.fastq.gz
D347-D340C10_trimmed.R1.fastq.gz D347-D340C10_trimmed.R2.fastq.gz
D347-D340C11_trimmed.R1.fastq.gz D347-D340C11_trimmed.R2.fastq.gz
D347-D340C13_trimmed.R1.fastq.gz D347-D340C13_trimmed.R2.fastq.gz
D347-D340C14_trimmed.R1.fastq.gz D347-D340C14_trimmed.R2.fastq.gz
D347-D340C16_trimmed.R1.fastq.gz D347-D340C16_trimmed.R2.fastq.gz
D347-D340C18_trimmed.R1.fastq.gz D347-D340C18_trimmed.R2.fastq.gz
D347-D340C19_trimmed.R1.fastq.gz D347-D340C19_trimmed.R2.fastq.gz
D347-D340C21_trimmed.R1.fastq.gz D347-D340C21_trimmed.R2.fastq.gz
D347-D340C22_trimmed.R1.fastq.gz D347-D340C22_trimmed.R2.fastq.gz
D347-D340C24_trimmed.R1.fastq.gz D347-D340C24_trimmed.R2.fastq.gz
D347-D340C26_trimmed.R1.fastq.gz D347-D340C26_trimmed.R2.fastq.gz
D851-D842C01_trimmed.R1.fastq.gz D851-D842C01_trimmed.R2.fastq.gz
D851-D842C02_trimmed.R1.fastq.gz D851-D842C02_trimmed.R2.fastq.gz
D851-D842C05_trimmed.R1.fastq.gz D851-D842C05_trimmed.R2.fastq.gz
D851-D842C06_trimmed.R1.fastq.gz D851-D842C06_trimmed.R2.fastq.gz
D851-D842C08_trimmed.R1.fastq.gz D851-D842C08_trimmed.R2.fastq.gz
D851-D842C09_trimmed.R1.fastq.gz D851-D842C09_trimmed.R2.fastq.gz
D851-D842C10_trimmed.R1.fastq.gz D851-D842C10_trimmed.R2.fastq.gz
D851-D842C11_trimmed.R1.fastq.gz D851-D842C11_trimmed.R2.fastq.gz
D851-D842C12_trimmed.R1.fastq.gz D851-D842C12_trimmed.R2.fastq.gz
D851-D842C13_trimmed.R1.fastq.gz D851-D842C13_trimmed.R2.fastq.gz
D851-D842C14_trimmed.R1.fastq.gz D851-D842C14_trimmed.R2.fastq.gz
D851-D842C15_trimmed.R1.fastq.gz D851-D842C15_trimmed.R2.fastq.gz
D851-D842C16_trimmed.R1.fastq.gz D851-D842C16_trimmed.R2.fastq.gz
D851-D842C17_trimmed.R1.fastq.gz D851-D842C17_trimmed.R2.fastq.gz
D851-D842C18_trimmed.R1.fastq.gz D851-D842C18_trimmed.R2.fastq.gz
D851-D842C19_trimmed.R1.fastq.gz D851-D842C19_trimmed.R2.fastq.gz
D851-D842C20_trimmed.R1.fastq.gz D851-D842C20_trimmed.R2.fastq.gz
D851-D842C21_trimmed.R1.fastq.gz D851-D842C21_trimmed.R2.fastq.gz
D851-D842C22_trimmed.R1.fastq.gz D851-D842C22_trimmed.R2.fastq.gz
D851-D842C23_trimmed.R1.fastq.gz D851-D842C23_trimmed.R2.fastq.gz
D851-D842C24_trimmed.R1.fastq.gz D851-D842C24_trimmed.R2.fastq.gz
D1371C40_trimmed_R1.fastq.gz
D1371C40_trimmed_R2.fastq.gz
D1371C41_trimmed_R1.fastq.gz
D1371C41_trimmed_R2.fastq.gz
D1371C42_trimmed_R1.fastq.gz
D1371C42_trimmed_R2.fastq.gz
D1371C43_trimmed_R1.fastq.gz
D1371C43_trimmed_R2.fastq.gz
Processed data files:
D387C101.H2AUb.csaw.bw
D387C102.H2AUb.csaw.bw
D387C103.H2AUb.csaw.bw
D387C104.H2AUb.csaw.bw
D387C105.H2AUb.csaw.bw
D387C106.H2AUb.csaw.bw
D387C107.H2AUb.csaw.bw
D387C108.H2AUb.csaw.bw
D387C109.H2AUb.csaw.bw

D387C110.H2AUb.csaw.bw
D387C131.H3K4me3.csaw.bw
D387C132.H3K4me3.csaw.bw
D387C133.H3K4me3.csaw.bw
D387C134.H3K4me3.csaw.bw
D387C135.H3K4me3.csaw.bw
D387C136.H3K4me3.csaw.bw
D387C137.H3K4me3.csaw.bw
D387C138.H3K4me3.csaw.bw
D387C139.H3K4me3.csaw.bw
D387C140.H3K4me3.csaw.bw
D127C73.H3K27me2.csaw.bw
D127C74.H3K27me2.csaw.bw
D127C75.H3K27me2.csaw.bw
D127C76.H3K27me2.csaw.bw
D127C77.H3K27me2.csaw.bw
D127C78.H3K27me2.csaw.bw
D127C79.H3K27me2.csaw.bw
D127C80.H3K27me2.csaw.bw
D127C81.H3K27me2.csaw.bw
D127C82.H3K27me2.csaw.bw
D127C53.H3K27me3.csaw.bw
D127C54.H3K27me3.csaw.bw
D127C55.H3K27me3.csaw.bw
D127C56.H3K27me3.csaw.bw
D127C57.H3K27me3.csaw.bw
D127C58.H3K27me3.csaw.bw
D127C59.H3K27me3.csaw.bw
D127C60.H3K27me3.csaw.bw
D127C61.H3K27me3.csaw.bw
D127C62.H3K27me3.csaw.bw
D127C63.H3K27Ac.csaw.bw
D127C64.H3K27Ac.csaw.bw
D127C65.H3K27Ac.csaw.bw
D127C66.H3K27Ac.csaw.bw
D127C67.H3K27Ac.csaw.bw
D127C68.H3K27Ac.csaw.bw
D127C69.H3K27Ac.csaw.bw
D127C70.H3K27Ac.csaw.bw
D127C71.H3K27Ac.csaw.bw
D127C72.H3K27Ac.csaw.bw
D387C121.H3K36me3.csaw.bw
D387C122.H3K36me3.csaw.bw
D387C123.H3K36me3.csaw.bw
D387C124.H3K36me3.csaw.bw
D387C125.H3K36me3.csaw.bw
D387C126.H3K36me3.csaw.bw
D387C127.H3K36me3.csaw.bw
D387C128.H3K36me3.csaw.bw
D387C129.H3K36me3.csaw.bw
D387C130.H3K36me3.csaw.bw
D387C153.H3K27me3.csaw.bw
D387C154.H3K27me3.csaw.bw
D387C155.H3K27me3.csaw.bw
D387C156.H3K27me3.csaw.bw
D387C157.H3K27me3.csaw.bw
D387C158.H3K27me3.csaw.bw
D387C160.H3K27me3.csaw.bw
D387C161.H3K27me3.csaw.bw
D387C162.H3K27me3.csaw.bw
D387C163.H3K27me3.csaw.bw
D387C164.H3K27me3.csaw.bw
D387C165.H3K27me3.csaw.bw
D233C05.H3K27me3.csaw.bw
D233C06.H3K27me3.csaw.bw
D256-D251C26.H3K27me3.csaw.bw
D256-D251C27.H3K27me3.csaw.bw
D256-D251C29.H3K27me3.csaw.bw
D256-D251C30.H3K27me3.csaw.bw
D233C14.H3K27me3.csaw.bw
D233C15.H3K27me3.csaw.bw
D233C17.H3K27me3.csaw.bw
D233C18.H3K27me3.csaw.bw
D233C20.H3K27me3.csaw.bw
D233C21.H3K27me3.csaw.bw
D233C26.H3K27me3.csaw.bw

Methodology

Replicates

Two independent biological replicates were performed for each ChIP-seq experiment. Replicates were consistently in agreement, with one notable exception highlighted in the manuscript (Supplementary Fig. 4e, patient 23).

Sequencing depth

D387C101.H2AUb.csaw.bw Total reads: 9453800 Uniquely mapped reads: 5788440 Length of reads: 100 Paired-end
D387C102.H2AUb.csaw.bw Total reads: 9131124 Uniquely mapped reads: 5566834 Length of reads: 100 Paired-end
D387C103.H2AUb.csaw.bw Total reads: 15468040 Uniquely mapped reads: 11005200 Length of reads: 100 Paired-end
D387C104.H2AUb.csaw.bw Total reads: 21855080 Uniquely mapped reads: 15480468 Length of reads: 100 Paired-end
D387C105.H2AUb.csaw.bw Total reads: 16504168 Uniquely mapped reads: 11580602 Length of reads: 100 Paired-end
D387C106.H2AUb.csaw.bw Total reads: 15702042 Uniquely mapped reads: 10527046 Length of reads: 100 Paired-end
D387C107.H2AUb.csaw.bw Total reads: 15223858 Uniquely mapped reads: 10775148 Length of reads: 100 Paired-end
D387C108.H2AUb.csaw.bw Total reads: 9622762 Uniquely mapped reads: 5228762 Length of reads: 100 Paired-end
D387C109.H2AUb.csaw.bw Total reads: 12241460 Uniquely mapped reads: 8818516 Length of reads: 100 Paired-end
D387C110.H2AUb.csaw.bw Total reads: 15831166 Uniquely mapped reads: 11312512 Length of reads: 100 Paired-end
D387C131.H3K4me3.csaw.bw Total reads: 18910990 Uniquely mapped reads: 14949102 Length of reads: 100 Paired-end
D387C132.H3K4me3.csaw.bw Total reads: 21520538 Uniquely mapped reads: 16648082 Length of reads: 100 Paired-end
D387C133.H3K4me3.csaw.bw Total reads: 18863546 Uniquely mapped reads: 13416454 Length of reads: 100 Paired-end
D387C134.H3K4me3.csaw.bw Total reads: 30613638 Uniquely mapped reads: 22583266 Length of reads: 100 Paired-end
D387C135.H3K4me3.csaw.bw Total reads: 21243880 Uniquely mapped reads: 16186980 Length of reads: 100 Paired-end
D387C136.H3K4me3.csaw.bw Total reads: 28293278 Uniquely mapped reads: 20029838 Length of reads: 100 Paired-end
D387C137.H3K4me3.csaw.bw Total reads: 19048210 Uniquely mapped reads: 14477262 Length of reads: 100 Paired-end
D387C138.H3K4me3.csaw.bw Total reads: 23259902 Uniquely mapped reads: 18055368 Length of reads: 100 Paired-end
D387C139.H3K4me3.csaw.bw Total reads: 20997670 Uniquely mapped reads: 16228706 Length of reads: 100 Paired-end
D387C140.H3K4me3.csaw.bw Total reads: 22029524 Uniquely mapped reads: 16994140 Length of reads: 100 Paired-end
D127C73.H3K27me2.csaw.bw Total reads: 56153284 Uniquely mapped reads: 48449382 Length of reads: 100 Paired-end
D127C74.H3K27me2.csaw.bw Total reads: 61431988 Uniquely mapped reads: 52467932 Length of reads: 100 Paired-end
D127C75.H3K27me2.csaw.bw Total reads: 44561646 Uniquely mapped reads: 38042692 Length of reads: 100 Paired-end
D127C76.H3K27me2.csaw.bw Total reads: 55133612 Uniquely mapped reads: 46598344 Length of reads: 100 Paired-end
D127C77.H3K27me2.csaw.bw Total reads: 46723060 Uniquely mapped reads: 36543128 Length of reads: 100 Paired-end
D127C78.H3K27me2.csaw.bw Total reads: 46034216 Uniquely mapped reads: 37401596 Length of reads: 100 Paired-end
D127C79.H3K27me2.csaw.bw Total reads: 57145268 Uniquely mapped reads: 49018664 Length of reads: 100 Paired-end
D127C80.H3K27me2.csaw.bw Total reads: 55508162 Uniquely mapped reads: 47563642 Length of reads: 100 Paired-end
D127C81.H3K27me2.csaw.bw Total reads: 52204662 Uniquely mapped reads: 37883462 Length of reads: 100 Paired-end
D127C82.H3K27me2.csaw.bw Total reads: 47559424 Uniquely mapped reads: 38082822 Length of reads: 100 Paired-end
D127C53.H3K27me3.csaw.bw Total reads: 51255854 Uniquely mapped reads: 41486896 Length of reads: 100 Paired-end
D127C54.H3K27me3.csaw.bw Total reads: 56437692 Uniquely mapped reads: 46194320 Length of reads: 100 Paired-end
D127C55.H3K27me3.csaw.bw Total reads: 51028118 Uniquely mapped reads: 39462932 Length of reads: 100 Paired-end
D127C56.H3K27me3.csaw.bw Total reads: 52883674 Uniquely mapped reads: 41937520 Length of reads: 100 Paired-end
D127C57.H3K27me3.csaw.bw Total reads: 94968962 Uniquely mapped reads: 78910504 Length of reads: 100 Paired-end
D127C58.H3K27me3.csaw.bw Total reads: 72177534 Uniquely mapped reads: 59173698 Length of reads: 100 Paired-end
D127C59.H3K27me3.csaw.bw Total reads: 46971506 Uniquely mapped reads: 36464682 Length of reads: 100 Paired-end
D127C60.H3K27me3.csaw.bw Total reads: 36729808 Uniquely mapped reads: 28221356 Length of reads: 100 Paired-end
D127C61.H3K27me3.csaw.bw Total reads: 39355654 Uniquely mapped reads: 30263188 Length of reads: 100 Paired-end
D127C62.H3K27me3.csaw.bw Total reads: 41018948 Uniquely mapped reads: 30095804 Length of reads: 100 Paired-end
D127C63.H3K27Ac.csaw.bw Total reads: 39573210 Uniquely mapped reads: 32494930 Length of reads: 100 Paired-end
D127C64.H3K27Ac.csaw.bw Total reads: 36257902 Uniquely mapped reads: 30143134 Length of reads: 100 Paired-end
D127C65.H3K27Ac.csaw.bw Total reads: 41799796 Uniquely mapped reads: 35839578 Length of reads: 100 Paired-end
D127C66.H3K27Ac.csaw.bw Total reads: 56150946 Uniquely mapped reads: 47832358 Length of reads: 100 Paired-end
D127C67.H3K27Ac.csaw.bw Total reads: 63622364 Uniquely mapped reads: 52849510 Length of reads: 100 Paired-end
D127C68.H3K27Ac.csaw.bw Total reads: 46542542 Uniquely mapped reads: 39358744 Length of reads: 100 Paired-end
D127C69.H3K27Ac.csaw.bw Total reads: 46860264 Uniquely mapped reads: 38944094 Length of reads: 100 Paired-end
D127C70.H3K27Ac.csaw.bw Total reads: 46037564 Uniquely mapped reads: 38403646 Length of reads: 100 Paired-end
D127C71.H3K27Ac.csaw.bw Total reads: 45516202 Uniquely mapped reads: 38248530 Length of reads: 100 Paired-end
D127C72.H3K27Ac.csaw.bw Total reads: 46619426 Uniquely mapped reads: 39701270 Length of reads: 100 Paired-end
D387C121.H3K36me3.csaw.bw Total reads: 14937132 Uniquely mapped reads: 9411784 Length of reads: 100 Paired-end
D387C122.H3K36me3.csaw.bw Total reads: 16540190 Uniquely mapped reads: 7225764 Length of reads: 100 Paired-end
D387C123.H3K36me3.csaw.bw Total reads: 19121118 Uniquely mapped reads: 12406410 Length of reads: 100 Paired-end
D387C124.H3K36me3.csaw.bw Total reads: 17204696 Uniquely mapped reads: 10711112 Length of reads: 100 Paired-end
D387C125.H3K36me3.csaw.bw Total reads: 24554976 Uniquely mapped reads: 16432212 Length of reads: 100 Paired-end
D387C126.H3K36me3.csaw.bw Total reads: 17551554 Uniquely mapped reads: 10408122 Length of reads: 100 Paired-end
D387C127.H3K36me3.csaw.bw Total reads: 27130382 Uniquely mapped reads: 15280964 Length of reads: 100 Paired-end
D387C128.H3K36me3.csaw.bw Total reads: 22314674 Uniquely mapped reads: 13191728 Length of reads: 100 Paired-end
D387C129.H3K36me3.csaw.bw Total reads: 19292190 Uniquely mapped reads: 10797232 Length of reads: 100 Paired-end
D387C130.H3K36me3.csaw.bw Total reads: 19182702 Uniquely mapped reads: 11646306 Length of reads: 100 Paired-end
D387C153.H3K27me3.csaw.bw Total reads: 22281402 Uniquely mapped reads: 16090346 Length of reads: 100 Paired-end
D387C154.H3K27me3.csaw.bw Total reads: 22707098 Uniquely mapped reads: 17681594 Length of reads: 100 Paired-end
D387C155.H3K27me3.csaw.bw Total reads: 18746382 Uniquely mapped reads: 13053594 Length of reads: 100 Paired-end
D387C156.H3K27me3.csaw.bw Total reads: 21627318 Uniquely mapped reads: 15511484 Length of reads: 100 Paired-end
D387C157.H3K27me3.csaw.bw Total reads: 22715344 Uniquely mapped reads: 15982544 Length of reads: 100 Paired-end
D387C158.H3K27me3.csaw.bw Total reads: 27233568 Uniquely mapped reads: 20704088 Length of reads: 100 Paired-end
D387C160.H3K27me3.csaw.bw Total reads: 22037760 Uniquely mapped reads: 16442552 Length of reads: 100 Paired-end
D387C161.H3K27me3.csaw.bw Total reads: 24829810 Uniquely mapped reads: 17369064 Length of reads: 100 Paired-end
D387C162.H3K27me3.csaw.bw Total reads: 29691478 Uniquely mapped reads: 21370504 Length of reads: 100 Paired-end
D387C163.H3K27me3.csaw.bw Total reads: 21006738 Uniquely mapped reads: 14793768 Length of reads: 100 Paired-end

D387C164.H3K27me3.csaw.bw Total reads: 24861316 Uniquely mapped reads: 17789244 Length of reads: 100 Paired-end
D387C165.H3K27me3.csaw.bw Total reads: 17004464 Uniquely mapped reads: 10087910 Length of reads: 100 Paired-end
D233C05.H3K27me3.csaw.bw Total reads: 78960572 Uniquely mapped reads: 70745967 Length of reads: 100 Paired-end
D233C06.H3K27me3.csaw.bw Total reads: 54703034 Uniquely mapped reads: 51258225 Length of reads: 100 Paired-end
D256-D251C26.H3K27me3.csaw.bw Total reads: 146415134 Uniquely mapped reads: 138744235 Length of reads: 100 Paired-end
D256-D251C27.H3K27me3.csaw.bw Total reads: 52360472 Uniquely mapped reads: 48598844 Length of reads: 100 Paired-end
D256-D251C29.H3K27me3.csaw.bw Total reads: 51660834 Uniquely mapped reads: 48588214 Length of reads: 100 Paired-end
D256-D251C30.H3K27me3.csaw.bw Total reads: 51927496 Uniquely mapped reads: 48356107 Length of reads: 100 Paired-end
D233C14.H3K27me3.csaw.bw Total reads: 64189952 Uniquely mapped reads: 60258165 Length of reads: 100 Paired-end
D233C15.H3K27me3.csaw.bw Total reads: 66085108 Uniquely mapped reads: 62400407 Length of reads: 100 Paired-end
D233C17.H3K27me3.csaw.bw Total reads: 69723646 Uniquely mapped reads: 65483067 Length of reads: 100 Paired-end
D233C18.H3K27me3.csaw.bw Total reads: 53324892 Uniquely mapped reads: 49262275 Length of reads: 100 Paired-end
D233C20.H3K27me3.csaw.bw Total reads: 47062570 Uniquely mapped reads: 44191283 Length of reads: 100 Paired-end
D233C21.H3K27me3.csaw.bw Total reads: 52069664 Uniquely mapped reads: 48928014 Length of reads: 100 Paired-end
D233C26.H3K27me3.csaw.bw Total reads: 52034316 Uniquely mapped reads: 48914381 Length of reads: 100 Paired-end
D233C27.H3K27me3.csaw.bw Total reads: 61778394 Uniquely mapped reads: 57812989 Length of reads: 100 Paired-end
D256-D251C11.H3K27me3.csaw.bw Total reads: 52392360 Uniquely mapped reads: 48689429 Length of reads: 100 Paired-end
D256-D251C12.H3K27me3.csaw.bw Total reads: 54558642 Uniquely mapped reads: 47490917 Length of reads: 100 Paired-end
D256-D251C17.H3K27me3.csaw.bw Total reads: 52405696 Uniquely mapped reads: 48754044 Length of reads: 100 Paired-end
D256-D251C18.H3K27me3.csaw.bw Total reads: 52967302 Uniquely mapped reads: 50461032 Length of reads: 100 Paired-end
D256-D251C20.H3K27me3.csaw.bw Total reads: 53033800 Uniquely mapped reads: 49331678 Length of reads: 100 Paired-end
D256-D251C21.H3K27me3.csaw.bw Total reads: 50661918 Uniquely mapped reads: 46853524 Length of reads: 100 Paired-end
D256-D251C23.H3K27me3.csaw.bw Total reads: 52381046 Uniquely mapped reads: 47420881 Length of reads: 100 Paired-end
D256-D251C24.H3K27me3.csaw.bw Total reads: 52113602 Uniquely mapped reads: 49132700 Length of reads: 100 Paired-end
D164C07.H3K27me3.csaw.bw Total reads: 114013792 Uniquely mapped reads: 102590860 Length of reads: 100 Paired-end
D164C09.H3K27me3.csaw.bw Total reads: 80776432 Uniquely mapped reads: 75083566 Length of reads: 100 Paired-end
D330C05.H3K27me3.csaw.bw Total reads: 50152260 Uniquely mapped reads: 47144298 Length of reads: 100 Paired-end
D330C06.H3K27me3.csaw.bw Total reads: 51176692 Uniquely mapped reads: 48026537 Length of reads: 100 Paired-end
D233C23.H3K27me3.csaw.bw Total reads: 59275628 Uniquely mapped reads: 55480506 Length of reads: 100 Paired-end
D330C07.H3K27me3.csaw.bw Total reads: 35960604 Uniquely mapped reads: 32789918 Length of reads: 100 Paired-end
D233C08.H3K27me3.csaw.bw Total reads: 48232104 Uniquely mapped reads: 44658663 Length of reads: 100 Paired-end
D233C09.H3K27me3.csaw.bw Total reads: 59309980 Uniquely mapped reads: 55237597 Length of reads: 100 Paired-end
D330C12.H3K27me3.csaw.bw Total reads: 50184398 Uniquely mapped reads: 46994032 Length of reads: 100 Paired-end
D330C13.H3K27me3.csaw.bw Total reads: 39460954 Uniquely mapped reads: 37003732 Length of reads: 100 Paired-end
D330C15.H3K27me3.csaw.bw Total reads: 49329170 Uniquely mapped reads: 45152892 Length of reads: 100 Paired-end
D330C16.H3K27me3.csaw.bw Total reads: 43420298 Uniquely mapped reads: 39221011 Length of reads: 100 Paired-end
D330C17.H3K27me3.csaw.bw Total reads: 63655616 Uniquely mapped reads: 59857282 Length of reads: 100 Paired-end
D330C18.H3K27me3.csaw.bw Total reads: 34964218 Uniquely mapped reads: 32914169 Length of reads: 100 Paired-end
D164C17.H3K27me3.csaw.bw Total reads: 40563938 Uniquely mapped reads: 37428579 Length of reads: 100 Paired-end
D164C19.H3K27me3.csaw.bw Total reads: 22856492 Uniquely mapped reads: 18638103 Length of reads: 100 Paired-end
D256-D251C02.H3K27me3.csaw.bw Total reads: 51689308 Uniquely mapped reads: 48599870 Length of reads: 100 Paired-end
D256-D251C03.H3K27me3.csaw.bw Total reads: 53051380 Uniquely mapped reads: 49922925 Length of reads: 100 Paired-end
D330C19.H3K27me3.csaw.bw Total reads: 59783300 Uniquely mapped reads: 55877481 Length of reads: 100 Paired-end
D330C20.H3K27me3.csaw.bw Total reads: 64552144 Uniquely mapped reads: 60395338 Length of reads: 100 Paired-end
D330C21.H3K27me3.csaw.bw Total reads: 45752524 Uniquely mapped reads: 43064546 Length of reads: 100 Paired-end
D347-D340C02.H3K27me3.csaw.bw Total reads: 59504954 Uniquely mapped reads: 55667515 Length of reads: 100 Paired-end
D347-D340C03.H3K27me3.csaw.bw Total reads: 71064560 Uniquely mapped reads: 66645995 Length of reads: 100 Paired-end
D347-D340C05.H3K27me3.csaw.bw Total reads: 77656970 Uniquely mapped reads: 72818834 Length of reads: 100 Paired-end
D347-D340C07.H3K27me3.csaw.bw Total reads: 98355146 Uniquely mapped reads: 91100150 Length of reads: 100 Paired-end
D347-D340C08.H3K27me3.csaw.bw Total reads: 72540072 Uniquely mapped reads: 67331651 Length of reads: 100 Paired-end
D347-D340C10.H3K27me3.csaw.bw Total reads: 67848232 Uniquely mapped reads: 63732667 Length of reads: 100 Paired-end
D347-D340C11.H3K27me3.csaw.bw Total reads: 90140306 Uniquely mapped reads: 84665899 Length of reads: 100 Paired-end
D347-D340C13.H3K27me3.csaw.bw Total reads: 105828704 Uniquely mapped reads: 97807385 Length of reads: 100 Paired-end
D347-D340C14.H3K27me3.csaw.bw Total reads: 78260006 Uniquely mapped reads: 73600510 Length of reads: 100 Paired-end
D347-D340C16.H3K27me3.csaw.bw Total reads: 61321576 Uniquely mapped reads: 57263528 Length of reads: 100 Paired-end
D347-D340C18.H3K27me3.csaw.bw Total reads: 87874096 Uniquely mapped reads: 82898707 Length of reads: 100 Paired-end
D347-D340C19.H3K27me3.csaw.bw Total reads: 75482716 Uniquely mapped reads: 71218540 Length of reads: 100 Paired-end
D347-D340C21.H3K27me3.csaw.bw Total reads: 100847322 Uniquely mapped reads: 94738451 Length of reads: 100 Paired-end
D347-D340C22.H3K27me3.csaw.bw Total reads: 71878892 Uniquely mapped reads: 67501575 Length of reads: 100 Paired-end
D347-D340C24.H3K27me3.csaw.bw Total reads: 55252760 Uniquely mapped reads: 46772288 Length of reads: 100 Paired-end
D347-D340C26.H3K27me3.csaw.bw Total reads: 62875990 Uniquely mapped reads: 57669833 Length of reads: 100 Paired-end
D851-D842C01.H3K27Ac.csaw.bw Total reads: 61399980 Uniquely mapped reads: 51926396 Length of reads: 100 Paired-end
D851-D842C02.H3K27Ac.csaw.bw Total reads: 31693520 Uniquely mapped reads: 27053717 Length of reads: 100 Paired-end
D851-D842C05.H3K27Ac.csaw.bw Total reads: 48543560 Uniquely mapped reads: 38322729 Length of reads: 100 Paired-end
D851-D842C06.H3K27Ac.csaw.bw Total reads: 30894962 Uniquely mapped reads: 29408876 Length of reads: 100 Paired-end
D851-D842C08.H3K27Ac.csaw.bw Total reads: 39694276 Uniquely mapped reads: 37425432 Length of reads: 100 Paired-end
D851-D842C09.H3K27Ac.csaw.bw Total reads: 38293520 Uniquely mapped reads: 32117360 Length of reads: 100 Paired-end
D851-D842C10.H3K27Ac.csaw.bw Total reads: 33893448 Uniquely mapped reads: 31915147 Length of reads: 100 Paired-end
D851-D842C11.H3K27Ac.csaw.bw Total reads: 49308136 Uniquely mapped reads: 46557045 Length of reads: 100 Paired-end
D851-D842C12.H3K27Ac.csaw.bw Total reads: 31683528 Uniquely mapped reads: 29997303 Length of reads: 100 Paired-end
D851-D842C13.H3K27Ac.csaw.bw Total reads: 37354800 Uniquely mapped reads: 35462224 Length of reads: 100 Paired-end
D851-D842C14.H3K27Ac.csaw.bw Total reads: 29990584 Uniquely mapped reads: 26758938 Length of reads: 100 Paired-end
D851-D842C15.H3K27Ac.csaw.bw Total reads: 38225318 Uniquely mapped reads: 36095109 Length of reads: 100 Paired-end
D851-D842C16.H3K27Ac.csaw.bw Total reads: 37837968 Uniquely mapped reads: 35682679 Length of reads: 100 Paired-end
D851-D842C17.H3K27Ac.csaw.bw Total reads: 55289360 Uniquely mapped reads: 52017296 Length of reads: 100 Paired-end
D851-D842C18.H3K27Ac.csaw.bw Total reads: 28701524 Uniquely mapped reads: 24281724 Length of reads: 100 Paired-end

D233C27.H3K27me3.csaw.bw
D256-D251C11.H3K27me3.csaw.bw
D256-D251C12.H3K27me3.csaw.bw
D256-D251C17.H3K27me3.csaw.bw
D256-D251C18.H3K27me3.csaw.bw
D256-D251C20.H3K27me3.csaw.bw
D256-D251C21.H3K27me3.csaw.bw
D256-D251C23.H3K27me3.csaw.bw
D256-D251C24.H3K27me3.csaw.bw
D164C07.H3K27me3.csaw.bw
D164C09.H3K27me3.csaw.bw
D330C05.H3K27me3.csaw.bw
D330C06.H3K27me3.csaw.bw
D233C23.H3K27me3.csaw.bw
D330C07.H3K27me3.csaw.bw
D233C08.H3K27me3.csaw.bw
D233C09.H3K27me3.csaw.bw
D330C12.H3K27me3.csaw.bw
D330C13.H3K27me3.csaw.bw
D330C15.H3K27me3.csaw.bw
D330C16.H3K27me3.csaw.bw
D330C17.H3K27me3.csaw.bw
D330C18.H3K27me3.csaw.bw
D164C17.H3K27me3.csaw.bw
D164C19.H3K27me3.csaw.bw
D256-D251C02.H3K27me3.csaw.bw
D256-D251C03.H3K27me3.csaw.bw
D330C19.H3K27me3.csaw.bw
D330C20.H3K27me3.csaw.bw
D330C21.H3K27me3.csaw.bw
D347-D340C02.H3K27me3.csaw.bw
D347-D340C03.H3K27me3.csaw.bw
D347-D340C05.H3K27me3.csaw.bw
D347-D340C07.H3K27me3.csaw.bw
D347-D340C08.H3K27me3.csaw.bw
D347-D340C10.H3K27me3.csaw.bw
D347-D340C11.H3K27me3.csaw.bw
D347-D340C13.H3K27me3.csaw.bw
D347-D340C14.H3K27me3.csaw.bw
D347-D340C16.H3K27me3.csaw.bw
D347-D340C18.H3K27me3.csaw.bw
D347-D340C19.H3K27me3.csaw.bw
D347-D340C21.H3K27me3.csaw.bw
D347-D340C22.H3K27me3.csaw.bw
D347-D340C24.H3K27me3.csaw.bw
D347-D340C26.H3K27me3.csaw.bw
D851-D842C01.H3K27Ac.csaw.bw
D851-D842C02.H3K27Ac.csaw.bw
D851-D842C05.H3K27Ac.csaw.bw
D851-D842C06.H3K27Ac.csaw.bw
D851-D842C08.H3K27Ac.csaw.bw
D851-D842C09.H3K27Ac.csaw.bw
D851-D842C10.H3K27Ac.csaw.bw
D851-D842C11.H3K27Ac.csaw.bw
D851-D842C12.H3K27Ac.csaw.bw
D851-D842C13.H3K27Ac.csaw.bw
D851-D842C14.H3K27Ac.csaw.bw
D851-D842C15.H3K27Ac.csaw.bw
D851-D842C16.H3K27Ac.csaw.bw
D851-D842C17.H3K27Ac.csaw.bw
D851-D842C18.H3K27Ac.csaw.bw
D851-D842C19.H3K27Ac.csaw.bw
D851-D842C20.H3K27Ac.csaw.bw
D851-D842C21.H3K27Ac.csaw.bw
D851-D842C22.H3K27Ac.csaw.bw
D851-D842C23.H3K27Ac.csaw.bw
D851-D842C24.H3K27Ac.csaw.bw
D1371C40.Suz12.csaw.bw
D1371C41.Suz12.csaw.bw
D1371C42.Suz12.csaw.bw
D1371C43.Suz12.csaw.bw

Genome browser session
(e.g. UCSC)

https://genome.ucsc.edu/s/laia_richart_gines/hg19_Romero_et_al
https://genome.ucsc.edu/s/laia_richart_gines/mm10_Romero_et_al_EZH2i
https://genome.ucsc.edu/s/laia_richart_gines/mm10_Romero_et_al

D851-D842C19.H3K27Ac.csaw.bw Total reads: 35639928 Uniquely mapped reads: 33820145 Length of reads: 100 Paired-end
D851-D842C20.H3K27Ac.csaw.bw Total reads: 40200902 Uniquely mapped reads: 33549650 Length of reads: 100 Paired-end
D851-D842C21.H3K27Ac.csaw.bw Total reads: 36598082 Uniquely mapped reads: 34729274 Length of reads: 100 Paired-end

Antibodies

Antibody Host Source Clone/identifier
H3K27me3 rabbit monoclonal CST C36B11
H3K27me2 mouse monoclonal Active Motif 324
H2Aub rabbit monoclonal CST 8240S
H3K27ac rabbit polyclonal Abcam Ab4729
H3K4me3 rabbit monoclonal CST C42D8
H3K36me3 rabbit polyclonal Abcam Ab9050
SUZ12 rabbit monoclonal CST D39F6

Peak calling parameters

Adapters and low_quality bases (<Q20) were removed from reads with TrimGalore (v0.4.0) and Cutadapt (v1.8.2). Trimmed reads were mapped to the mouse reference genome mm10 or to the human reference genome hg19, as appropriate, with Bowtie2 (v2.2.5) using default parameters. PCR duplicates were removed with Picard Tools MarkDuplicates (v1.97).
MEFs:
H3K27me3 and H3K27Ac peaks were called with MACS2 (v2.0.10) callpeak on deduplicated BAM files with the following parameters: -q 0.05 --broad --broad-cutoff 0.05. The CUT&RUN-seq experiments for H3K27me3 on Eed-KO cells were used as control for H3K27me3 peak calling. CUT&RUN-Seq experiments performed with IgG were used as control for H3K27ac peak calling. After discarding peaks overlapping blacklisted regions (ref. 86 in manuscript), peaks lying less than 5 kb apart were merged using BEDtools merge (v2.2.2). To locate H3K27me2-rich regions, we used csaw to identify 10-kb, 5-kb, and 1-kb windows with an enrichment at least 2-fold higher than that of the background. The resulting collection of enriched bins was concatenated into one consensus set of regions, where bins within 5 kb were merged together.
Patient follicular lymphoma samples:
H3K27ac peaks were identified using the same MACS2 parameters as in iMEFs. Csaw was used to map H3K27me3-rich regions by first identifying 10-kb, 5-kb, and 1-kb windows with an enrichment at least 2-fold above background levels and then concatenating the resulting collection of bins. As with iMEFs, H3K27ac and H3K27me3 peaks less than 5 kb apart were merged together.

Data quality

Methods used to ensure data quality are detailed just above in "Peak calling parameters." All peaks fulfill the FDR 5% cutoff. Numbers of peaks called using MACS2 above 5-fold enrichment are as follows:
H3K27me3 in WT iMEFs: 498 of 53959 unmerged peaks
H3K27me3 in H3K27M iMEFs: 465 of 15984 unmerged peaks
H3K27ac in WT iMEFs: 11727 of 49069 unmerged peaks
H3K27ac in H3K27M iMEFs: 3287 of 56421 unmerged peaks
H3K27ac in Ezh2-KO iMEFs: 1746 of 51846 unmerged peaks
H3K27ac in Eed-KO iMEFs: 978 of 65433 unmerged peaks
H3K27ac in Ezh2-Y641F iMEFs: 14603 of 50750 unmerged peaks
H3K27ac in EZH2-WT/EZH2-mutant follicular lymphomas: 15628 of 238236 unmerged peaks

Software

All software used to analyze the ChIP-seq data is described just above in "Peak calling parameters."
